# A Wearable, Dual Closed‐loop Insulin Delivery System for Precision Diabetes Management

**DOI:** 10.1002/adma.202514945

**Published:** 2026-01-12

**Authors:** Xuecheng He, Wei Huang, Wensheng Lin, Binbin Cui, Xinyu Tian, Jing Bai, Dingyao Liu, Ivo Pang, Hao Huang, Shixian Lin, Jixiang Zhu, Jinqiang Wang, Shiming Zhang

**Affiliations:** ^1^ Department of Electrical and Electronic Engineering The University of Hong Kong Hong Kong SAR 999077 China; ^2^ School of Biomedical Engineering The University of Hong Kong Hong Kong SAR 999077 China; ^3^ The Fourth Affiliated Hospital of Guangzhou Medical University School of Biomedical Engineering Guangzhou Medical University Guangzhou 511436 China; ^4^ State Key Laboratory of Advanced Drug Delivery Systems Key Laboratory of Advanced Drug Delivery Systems of Zhejiang Province College of Pharmaceutical Sciences Zhejiang University Hangzhou 310058 China; ^5^ State Key Laboratory of Pharmaceutical Biotechnology The University of Hong Kong Hong Kong SAR 999077 China

**Keywords:** diabetic management, digital healthcare, dual closed‐loop, insulin, wearable AI

## Abstract

Effective blood glucose management is an increasing demand worldwide. Traditional solutions separate glucose detection and insulin delivery, which is less efficient compared to emerging closed‐loop wearable systems controlled by continuous glucose monitors (CGMs). However, CGM‐controlled systems raise new safety risks, as false CGMs readings can cause insulin overdose, which results in hypoglycemia and fatal consequences. This work proposes a concept of a dual closed‐loop insulin delivery system (DuoLoop) to mitigate the risk issue of CGM‐controlled systems. The first closed‐loop is automated insulin delivery controlled by CGM. The second closed‐loop is the controlled release of glucose‐responsive insulin (GRI), whose release rate depends on actual glucose levels. A customized algorithm is trained and embedded into the wearable CGMs for edge computing. The DuoLoop system shows improved safety in preliminary in vivo test (longer normoglycemia durations, 98.82% vs 92.10%), encouraging its deployment toward precision diabetes care.

## Graphical abstract

A wearable dual closed‐loop drug delivery system for diabetes management is presented, featuring an adaptive, on‐body trained edge AI algorithm for precise insulin delivery, which enhances therapeutic accuracy from 92.10% to 98.82%.

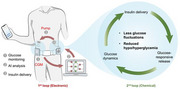

## 1 Introduction

Diabetes, a chronic global disease characterized by impaired insulin secretion and persistent hyperglycemia, may affect 643 million people worldwide by 2030.^[^ ^1^, ^2^, ^3^ ^]^ Traditionally, patients are treated with separate glucose detection and insulin delivery, which often leads to severe complications such as seizures, unconsciousness, or even death due to hypoglycemia.^[^ ^4^ ^]^ Currently, the emerging wearable biosensors,^[^ ^5^, ^6^, ^7^, ^8^, ^9^, ^10^, ^11^, ^12^ ^]^ such as the continuous glucose monitors (CGMs) controlled closed‐loop insulin delivery systems, have proven to be more effective in glycemic control over traditional methods.^[^ ^13^, ^14^, ^15^, ^16^, ^17^, ^18^ ^]^ The CGMs continuously track glucose levels, while the algorithm adjusts insulin release or suspension in response to real‐time glucose data. However, the current CGM‐controlled single closed‐loop (SinLoop) systems raise new safety risks. For example, false glucose readings can cause insulin overdose, which, in turn, results in hypoglycemia and fatal consequences.^[^ ^19^, ^20^ ^]^

To address the insulin overdose issue, glucose‐responsive insulins (GRIs) have been developed.^[^ ^21^, ^22^, ^23^, ^24^ ^]^ These GRIs emulate the function of β cells, with insulin release rates dynamically controlled by in vivo glucose levels in a chemically closed‐loop manner. Three primary glucose‐responsive mechanisms, including glucose‐binding protein,^[^ ^25^ ^]^ glucose oxidase,^[^ ^26^ ^]^ or phenylboronic acid,^[^ ^27^ ^]^ have been widely investigated for constructing GRIs. Although GRIs can spontaneously modulate insulin release to some extent, current administration methods, such as passive disposable patches,^[^ ^28^ ^]^ oral formulations,^[^ ^29^ ^]^ or manual injections,^[^ ^21^, ^30^ ^]^ lack integrated electronic systems for precise regulation. As a result, these approaches are still associated with risks of hyperglycemia or hypoglycemia.

As mentioned above, a CGM‐controlled system (electrical closed‐loop system) is effective in the automatic control of insulin release but carries a risk of overdose due to its intrinsic drawbacks. On the other hand, while GRIs (chemical closed‐loop systems) can help mitigate the risk of overdose to some extent, they still lack the capability for continuous and precise insulin delivery. We thus hypothesize that integrating CGM‐controlled systems with GRIs could establish an effective hybrid closed‐loop system, combining the strengths of both electrical and chemical closed‐loop approaches. However, this raises challenges in system engineering, as i) a customized algorithm and ii) a wearable hardware system are required to bring this concept to reality.^[^ ^31^ ^]^

**Figure 1:**
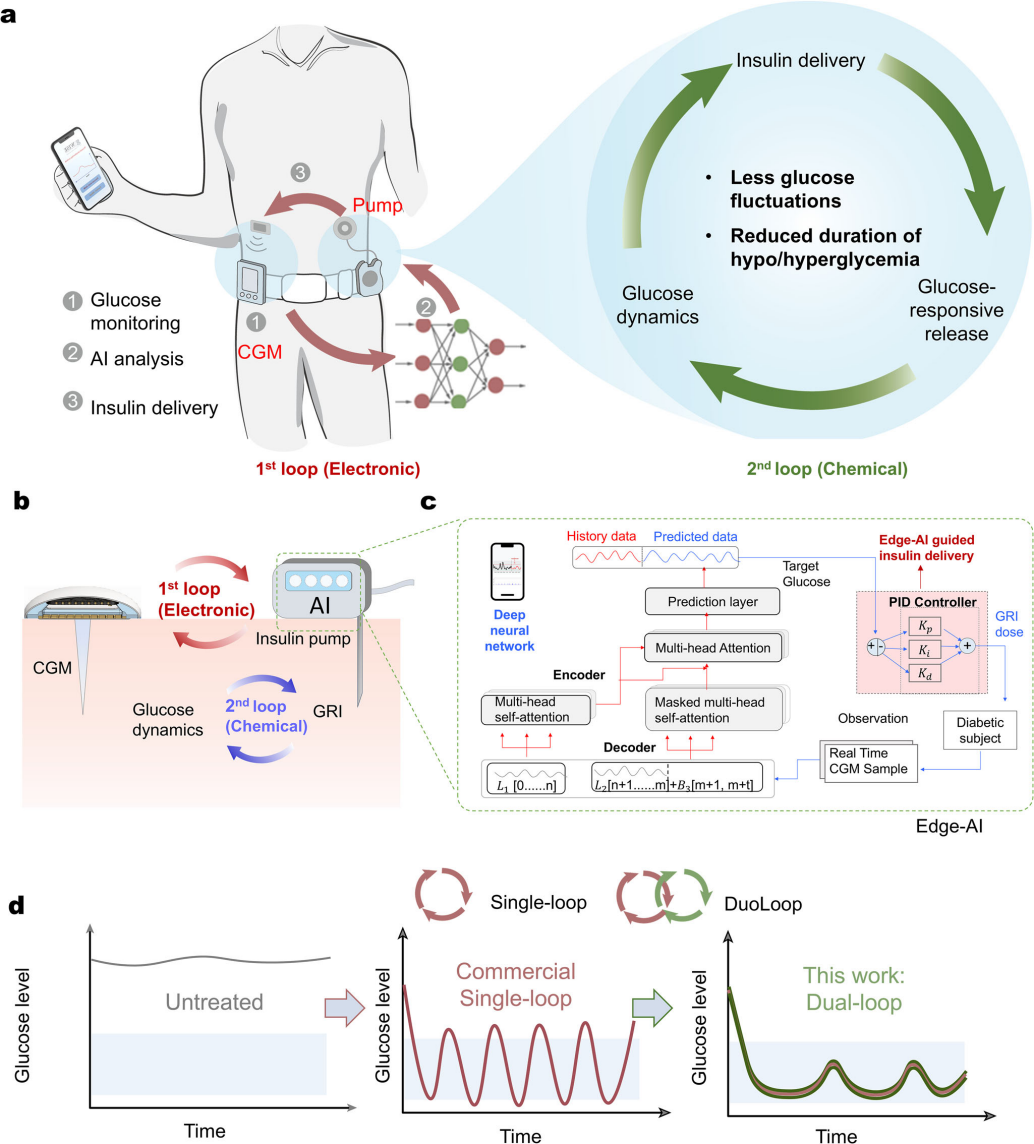
The overall concept of the proposed dual closed‐loop insulin delivery system (DuoLoop). a) Illustration of the key components of DuoLoop, including the CGM, algorithm, and insulin pump. b) Cross‐sectional illustration depicting the interrelationship among the components in DuoLoop. Schematics of the workflow and mechanism of c) quantized edge‐AI implementation of the dual closed‐loop system using a PID learning framework. d) Comparative schematic of glucose dynamics between the commercial SinLoop and the DuoLoop. Compared to the traditional single‐loop electrical closed‐loop system, DuoLoop demonstrates reduced glucose fluctuations and a lower incidence of hyperglycemia and hypoglycemia. K_p_​: Proportional gain, K_i_: Integral gain, K_d_: Derivative gain.

In this Article, we present a new “dual closed‐loop” wearable insulin delivery system (DuoLoop), which showed enhanced safety over traditional SinLoop systems. Our DuoLoop system includes the following three key components (**Figure** **1**a): (i) a CGM for the real‐time glucose analysis, (ii) a GRI, whose insulin delivery rate is governed by actual glucose levels, and (iii) a customized algorithm that is capable of predicting the glucose trend in the next 30 min for secured edge‐decision making. Given the limitations of traditional mathematical models in predicting glucose fluctuations accurately, we implemented a Transformer‐deep neural network (Figure 1b) to improve prediction accuracy. This system supports a proportional‐integral‐differential (PID)‐controlled GRI release mechanism (Figure 1c) with precise 30‐minute glucose predictions. The trained Transformer model showed its reliability (R^2^ = 98.03% and 97.83%, for the in‐domain and out‐of‐domain test sets, respectively) for precise glucose trend prediction. In vivo validations demonstrate that the DuoLoop achieves longer normoglycemia durations (98.82% vs 92.10%), reduced hyperglycemia (0.65% vs 3.89%), minimized hypoglycemia (0.52% vs 4.01%), and lower glucose fluctuations (standard deviation, SD: 2.44 vs 1.42, coefficient of variation, CV: 25.14 vs 41.22), over conventional SinLoop system (Figure 1d).

## 2 Results and Discussion

### 2.1 Real‐Time Glucose Monitoring with CGM

To implement the DuoLoop concept, we employed a commercial CGM for real‐time glucose monitoring. We also used our recently developed organic electrochemical transistor (OECT) based CGM as an alternative device for cross‐validation (Figure S1, Supporting Information). The OECT‐CGM shows a monotonic decrease in *I_ds_* (*I _drain‐source_*) across an extended glucose concentration range (0.4 to 30 mm, Figure S2, Supporting Information). This wide detection range allows the monitor of both hyperglycemic and hypoglycemic levels, which is beyond the sensing window of commercial CGMs (in the range of 2–25 mm). The glucose readout from the above CGMs was benchmarked with the blood glucose (BG) devices. The samples from healthy and streptozotocin (STZ)‐treated type 1 diabetic rats (Figure S3, Supporting Information) across various glycemic levels yielded a high coefficient of determination (R^2^ = 0.94, n = 31) between BG meter and the CGM readout (Figure S4, Supporting Information). This result aligns well with previous studies,^[^ ^32^, ^33^ ^]^ and suggests the reliability of the employed CGMs.

### 2.2 GRI Synthesis and Validation

**Figure 2:**
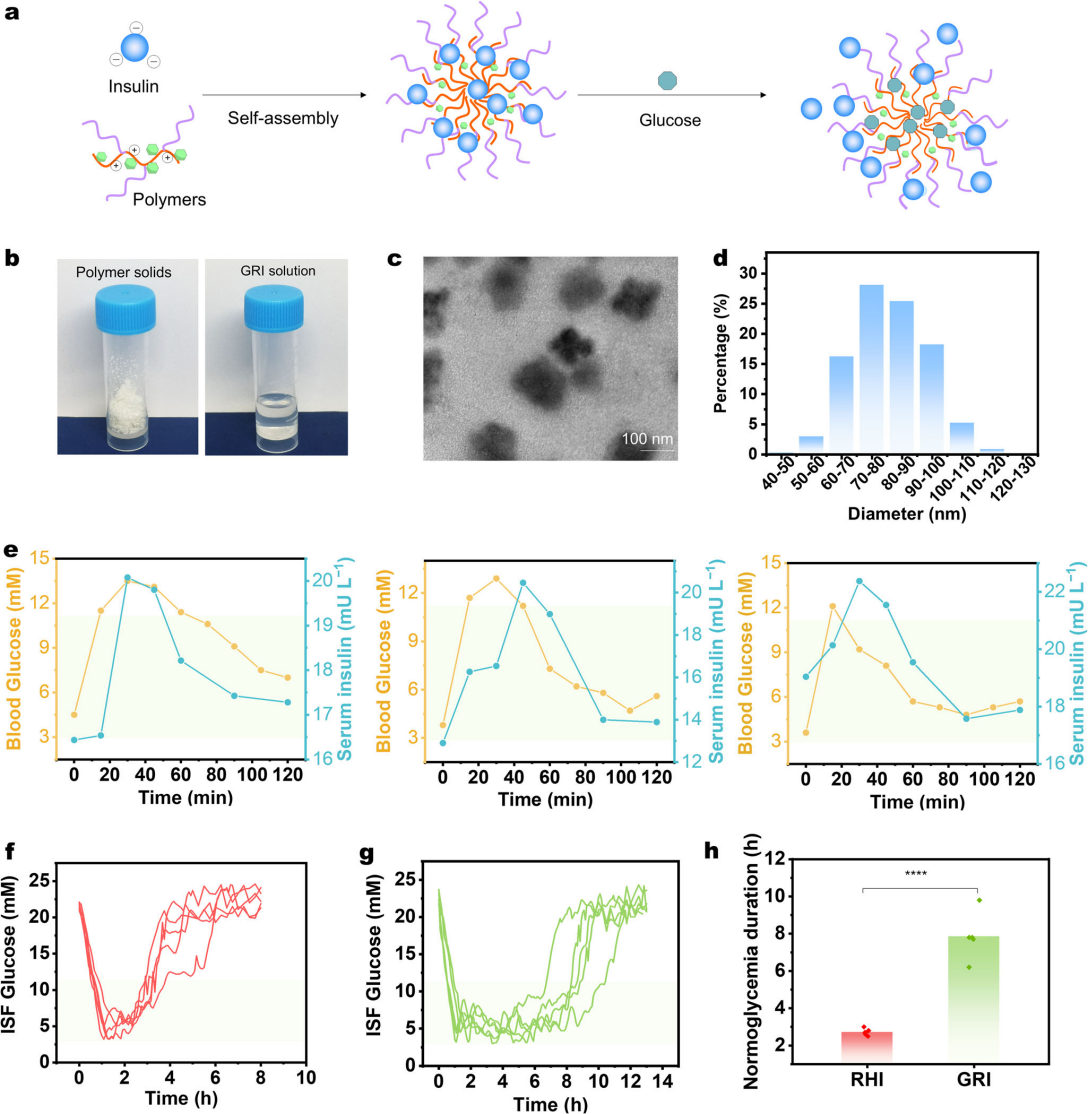
Synthesis and validation of GRI. a) Schematic representation of the glucose‐responsive mechanism of the GRI. b) The optical image of the polymer (solid) and polymer‐insulin complex (GRI, solution). c) The TEM images of polymer‐insulin complex nanomicelles. d) The size distribution of the GRI nanomicelles. e) BG‐triggered insulin release behavior following intraperitoneal glucose injection in diabetic rats. ISF glucose levels in diabetic rats after a single injection (25 U/kg) of f) RHI and g) GRI, along with h) the corresponding normoglycemia duration. Definitions of the ISF glucose concentrations are: hypoglycemia (<2.8 mm), normoglycemia (2.8–11.1 mm), and hyperglycemia (>11.1 mm) throughout this work.

The GRI was formulated as previously reported.^[^ ^21^ ^]^ In short, biodegradable poly‐L‐lysine (PLL) was modified with polyethylene glycol (PEG) and 4‐carboxy‐3‐fluorophenylboronic acid (FPBA) and obtained a glucose‐responsive polymer designated as PEG‐PLL‐FPBA (Figure S5, Supporting Information). PEG‐PLL‐FPBA attracts negatively charged recombinant human insulin (RHI) to form nanosized insulin complex (**Figure** **2**a). Under a high glucose level, FPBA binds to the glucose molecules rapidly, thus leading to a reduction in positive charge density and subsequent insulin release (Figure S6, Supporting Information). The PEG‐PLL‐FPBA polymer exhibited a white appearance, while the GRI solution remained clear and free of sediment (Figure 2b). Transmission electron microscopy (TEM, Figure 2c) and dynamic light scattering (DLS, Figure 2d) analyses demonstrated that the polymer‐insulin complex showing uniformly distributed nanomicelles with a diameter range around 100 nm. This nanoscale dimension ensures the injectability of GRI and prevents potential blockage of the needle/pump systems during subsequent delivery processes.

In vivo glucose‐triggered insulin release was further conducted to examine glucose‐responsive behavior. Diabetic rats received GRI via transcutaneous injection. Figure 2e shows three independent parallel experiments illustrating the dynamic relationship between interstitial fluid glucose and serum insulin (from GRI). Two hours after baseline, glucose (1.35 g kg^−1^) was administered to all diabetic rats (defined as 0 min in Figure 2e), causing a rapid rise in ISF glucose within about 15 min. This sharp increase effectively triggered a glucose‐dependent release of insulin, resulting in a serum insulin spike (21.0 ± 1.0 mU/L) at 30–45 min. The elevated GRI subsequently facilitated glucose clearance, reducing ISF glucose levels toward normoglycemia. As ISF glucose declined, negative feedback within the GRI system attenuated insulin release, leading to a gradual decrease in serum insulin concentrations between 30 and 60 min. The reproducibility of these glucose–insulin dynamics across three independent replicates highlights the robustness and reliability of the GRI‐mediated regulatory mechanism. To further understand the in vivo insulin release dynamics, we performed glucose tolerance tests 2 hours post‐GRI or RHI administration (Figure S7, Supporting Information). RHI‐treated rats showed a rapid BG increase and temporary decrease (less than 1 h), followed by obvious hyperglycemia. In contrast, GRI‐treated rats showed a delayed ISF glucose increase after glucose injection, followed by a rapid return to normoglycemia sustained for 5–6 hours. The above results confirm the effectiveness of GRI in dynamically regulating BG levels.

To determine the optimal dosing of GRI to be injected into diabetic rats, as shown in Figure S8 (Supporting Information), we further conducted dose‐response experiments with four RHI and GRI groups. Each GRI group maintained normoglycemia for a longer duration than the RHI group (with equivalent insulin doses). However, doses of 5 and 15 U/kg were insufficient to sustain normoglycemia, while 30 U/kg led tohypoglycemia. An optimal dose of 25 U/kg provided about 8 hours of normoglycemia without hypoglycemia and was thus selected as the maximum single‐injection dose for subsequent experiments unless specified otherwise. We further evaluated GRI's prolonged effect in five diabetic rats using 25 U/kg. The similarity is that ISF glucose dropped to normoglycemia within 0.5–1.5 h after injection of either RHI or GRI (25 U/kg). The major difference is that in RHI‐treated rats, ISF glucose returned to hyperglycemia within 3 hours due to limited retention (Figure 2f). In contrast, GRI achieved extended normoglycemia lasting 6–10 hours (Figure 2g,h). To calculate the duration of normoglycemia, we measured the time interval during which glucose levels remained between 2.8 and 11.1 mm.

### 2.3 Predictive Algorithm Development and Implementation

**Figure 3:**
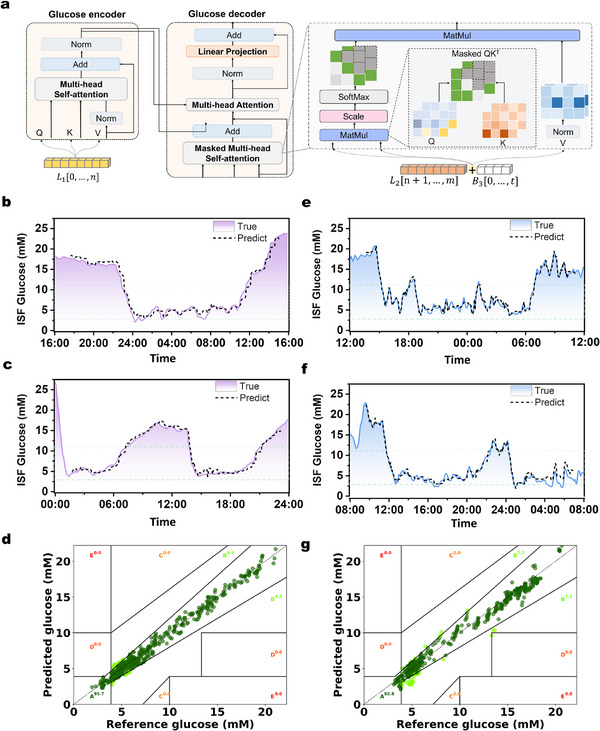
Algorithm development and validation for DuoLoop system. a) The structural scheme of the glucose prediction Transformer model. Experimental validation of the algorithm by comparing the predicted glycemia dynamics (using glucose prediction Transformer) with the glycemia dynamics in diabetic rats on the b,c) in‐domain test set and e,f) out‐of‐domain test set. Correlation analysis of the predicted versus actual ISF glucose level on d) the in‐domain test set g) out‐of‐domain test set.

An algorithm capable of predicting glucose trends is critical to further optimize the above GRI dosing and improve regulation efficiency.^[^ ^34^, ^35^ ^]^ To achieve this, we developed an end‐to‐end Transformer model with an Encoder‐Decoder structure. The Encoder includes two self‐attention layers, while the Decoder incorporates a masked self‐attention layer and an additional self‐attention layer, with an embedded hidden dimension of 128. As shown in **Figure** **3**a, we retain the Encoder‐Decoder structure of the Transformer and first embed the CGM‐recorded glucose signals, denoted as:

(1) $$L=L_{1}\;concat\;L_{2}\;concat\;B_{3}$$

where *L* ∈ *ℝ* ^(*m* × *d*)^ represents the historical CGM and injected insulin signals. *L*_1_ ∈ *ℝ* ^*n* × *d*^ corresponds to long‐range historical CGM data, *L*_2_ ∈ *ℝ* ^(*m* − *n*) × *d*^ corresponds to short‐range historical CGM data and *B_3_* ∈ *ℝ* ^6 × *d*^ denotes the blank position for 30 minutes prediction. The input data is processed using the standard absolute positional encoding in the Transformer. Additionally, to capture minute‐level patterns in glucose signals, we incorporate temporal encoding derived from CGM timestamps.

The input to the Encoder consists of the first *n* data points *L*_1_ from the historical encoded glucose sequence $L_{1}$. Following the design in previous work,^[^ ^36^ ^]^ the Encoder module learns long‐range information from the historical CGM signals, providing long‐term temporal dependencies for the prediction interval. The Decoder input is divided into two parts: the first part is the short‐range encoded CGM signals *L*_2_ corresponding to the last *m*–*n* data points of the historical glucose sequence *L*, and the second part consists of *t* empty encoding units, which serve as the prediction targets. Input sequences consist of 100 minutes of historical CGM data and insulin injection records. The Encoder processes 19 historical data sets and the current CGM blood glucose reading (*L*_1_, 100 minutes), while the Decoder uses the nearest 10 points of data (*L*_2_, 50 minutes) as input. The final 6 data points in the Decoder sequence are masked (*B*_3_, 30 minutes). The model outputs predicted glycemia dynamics for the next 30 minutes.

We validated the prediction accuracy of the proposed AI algorithm in a simulation and actual environment, where virtual diabetic patients and their simulated CGM data were generated over a 30‐day period using the UVA/Padova simulator.^[^ ^37^ ^]^ Glucose variations in these virtual patients were regulated using the conventional BASAL‐BOLUS insulin injection method.^[^ ^38^ ^]^ The data was then divided into a training set (60%), validation set (20%), and test set (20%). Training on virtual patients results in a rapid reduction in loss, indicating that the model's prediction error decreases as training progresses (Figure S9, Supporting Information),^[^ ^39^ ^]^ and the Transformer model's predictions closely match actual BG values for a randomly selected virtual patients, with a low root mean square error (RMSE) of 0.5 mm (Figure S10, Supporting Information), These results highlight the model's robust adaptability and strong generalizability for glucose prediction.

Following the simulator, we further assessed the algorithm in real diabetic rats through injection of GRI. Data from ten mice were collected over 10 days in an experimental setting, with blood glucose data obtained every five minutes and optimal GRI values recorded based on manual expertise. The dataset was divided into training, validation, and testing sets in a 6:2:2 ratio. Training on experimental diabetic rats also shows rapid loss reduction (Figure S11, Supporting Information). The diabetic rats were categorized into in‐domain (from the same rats used for training) and out‐of‐domain test sets (from different rats) groups to validate the predictive accuracy. The predicted glucose levels closely aligned with the actual measurements in both the in‐domain (Figure 3b,c) and out‐domain (Figure 3e,f) groups, with an RMSE of 1.03 mm. To further evaluate the reliability and safety of the glucose prediction system, we conducted a Clarke Error Grid (CEG) analysis, a widely used method for assessing glucose monitoring accuracy (Note S2, Supporting Information).^[^ ^40^ ^]^ The results clearly demonstrate that the majority of data points for both in‐domain and out‐of‐domain test sets fall within regions A (clinically accurate zone) and B (benign errors, clinically acceptable zone), indicating strong clinical reliability. Moreover, a linear correlation analysis between AI‐predicted and measured glucose levels revealed a high degree of association, with an R^2^ of 98.03% and 97.83% and a confidence level (buffer = 1) of 87.57% and 81.94% for the in‐domain and out‐of‐domain test sets, respectively. Bland–Altman analysis was performed to compare the predicted and measured glucose levels (Figure S12a, Supporting Information). The 95% limits of agreement included >94% of differences between the results from both in‐domain and out‐of‐domain. These findings collectively confirm the robustness and clinical applicability of the proposed algorithm, demonstrating its potential for reliable and accurate glucose monitoring in both controlled and variable conditions.

### 2.4 Closed‐Loop Insulin Injection Algorithm Implementation and Validation

Following the Transformer model, a PID closed‐loop control algorithm was constructed to integrate with the predictive model to optimize automatic GRI delivery. The PID algorithm uses proportional, integral, and derivative terms to model physiological insulin release,^[^ ^41^ ^]^ with the following formula:

(2) $$I_{t}=K_{p}\left(G_{t}-G_{B}\right)+K_{i}\int \left(G_{t}-G_{B}\right)dt+K_{d}\frac{dG}{dt}$$

where *G_B_* represents the target glucose level. The proportional term *K_p_* (*G_t_‐G_B_*) measures the linear difference between the current and target blood glucose, while the integral term *K_i_∫*(*G_t_‐G_B_*)*dt* corrects cumulative errors in real‐time, dynamically adjusting GRI predictions. The derivative term $K_{d}\frac{dG}{dt}$ captures glucose trends, allowing the PID system to estimate *dG*/*dt* = (*G_t_* − *G*_*t* − 1_)/5  min. This method relies entirely on past and current measurements, which may not fully capture future glucose trends. Since it only utilizes historical data, it cannot accurately reflect the true rate of change (derivative) at the current time point, as any unexpected or rapid fluctuations in glucose levels may not be adequately represented by past observations alone. In our proposed approach, we leverage the Transformer model's ability to predict future glucose levels with high precision. Specifically, we enhance the derivative term calculation by predicting the glucose value at the next time step, *G*_*t* + 1_, and using it to compute $\frac{dG}{dt}=\left(G_{t+1}-G_{t-1}\right)/\left(2\times 5\right)$ min. This adjustment allows the PID system to account for future glucose trends, providing a more precise and forward‐looking assessment of glucose dynamics. The Transformer model was also compressed to one‐fourth its parameter size and deployed on cell phones,^[^ ^42^ ^]^ enabling real‐time online processing (Table S1, Supporting Information). Similarly, a simulated closed‐loop insulin injection was conducted on ten virtual patients, yielding promising treatment outcomes (mean glucose level: 6.0 mm, median glucose level: 6.1 mm, normoglycemia percentage: 98.2%; Figure S13, Supporting Information). We also summarized the average Time‐in‐Range (TIR) percentages for virtual patients with and without the Transformer‐based predictions (Table S2, Supporting Information). The results indicate that the inclusion of the Transformer significantly improves TIR, with an average of 98.2% compared to 86.9% without the Transformer. This improvement highlights the Transformer model's ability to predict future data effectively, allowing the PID controller to make more informed decisions and achieve better control over blood glucose levels.

**Figure 4:**
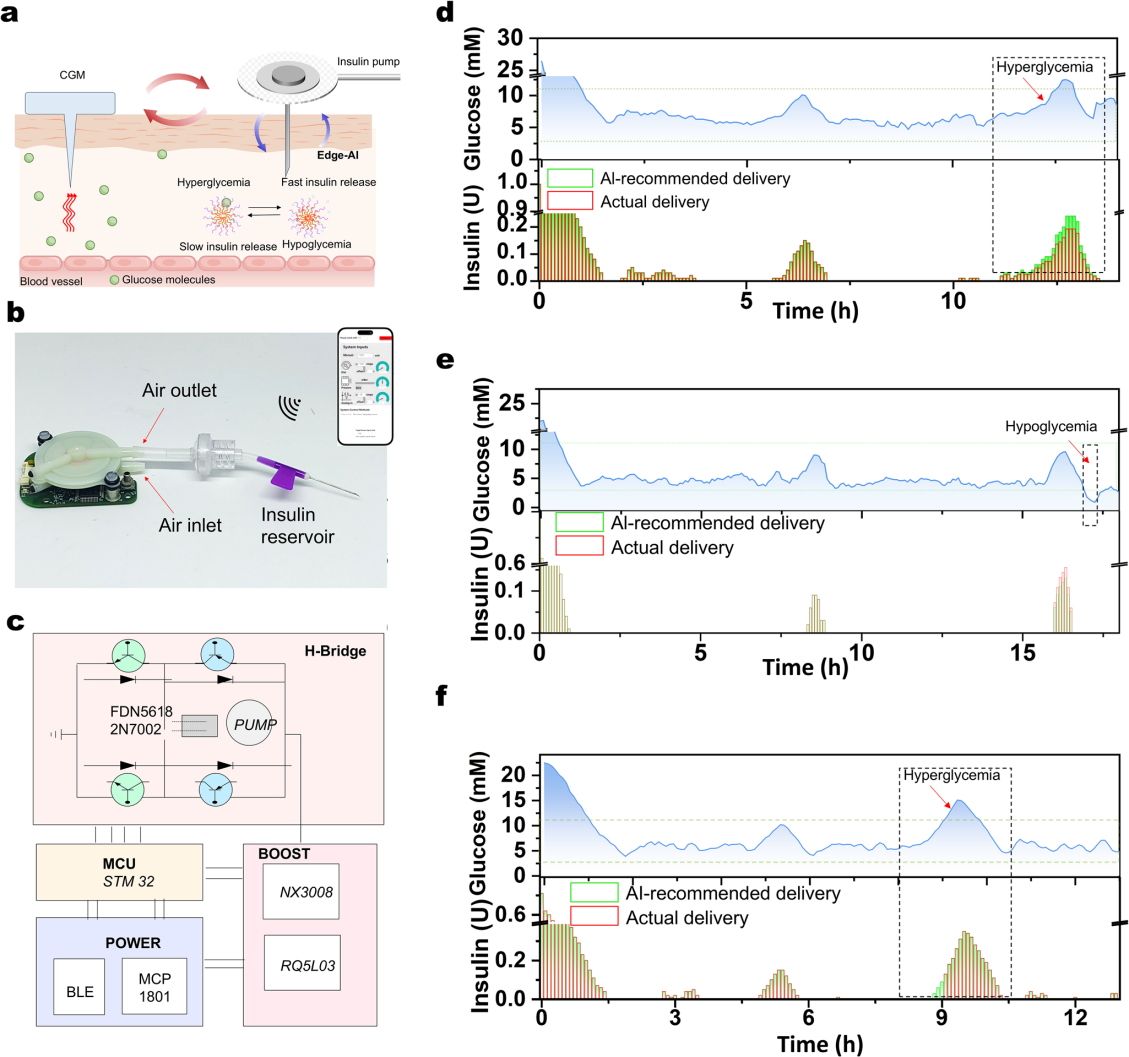
Algorithm implementation and validation of the DuoLoop system. a) The overall outline and layout of DuoLoop. b) Optical image of the semi‐customized insulin pump. Inset: user interface of the insulin pump control system. c) The functional block diagram highlighting the major electronic components. Experimental validation of the algorithm by comparison of glucose level under algorithm‐recommended GRI delivery dose (d) reduced dose; (e) increased dose) and timing f) versus that under non‐algorithmic dosing and timing conditions.

To implement a fully autonomous, algorithm‐controlled insulin delivery system, we constructed a miniature pump based on a pristine commercial product (**Figure** **4**a–c; Figure S14, Supporting Information) and integrated several key enhancements. First, we incorporated Bluetooth connectivity for wireless data reception (CGM prediction) and signal transmission (pump control). Second, we attached a catheter with a reservoir and syringe needle, enabling precise insulin storage and delivery. Third, the PID algorithm was deployed on the pump, facilitating real‐time, edge‐based GRI closed‐loop control. Upon receiving CGM prediction data via Bluetooth, the system anticipates future glucose trends and dynamically regulates insulin delivery. The integrated design allows for wireless operation via a user interface to adjust the power and insulin delivery rate (Figure S15a, Supporting Information), which can maintain optimized glucose levels of diabetic objects without disrupting their daily activities. The actual solution volumes showed good agreement with the set values (Figure S15b, Supporting Information), meeting the requirements for accurate insulin delivery.

To assess the accuracy and effectiveness of the PID closed‐loop control algorithm, we intentionally altered both the recommended insulin dose and injection timing and compared the glucose level under algorithm‐recommended GRI delivery dose and timing versus that under non‐algorithmic dosing and timing conditions using the above pump. First, an initial injection was administered to lower glycemia to a comparable normoglycemic level, allowing a clearer comparison of glycemic dynamics following subsequent injections. The algorithm‐guided insulin delivery kept glucose levels within the normoglycemic range (Figure 4d–f), while intentionally reduced and increased insulin doses caused hyperglycemia (Figure 4d) and hypoglycemia (Figure 4e), respectively. Briefly, in Figure 4d, the ISF glucose concentration rose at around the 11^th^ hour, at which point the algorithm began calculating the recommended insulin dose. We deliberately decreased the GRI dose to 80% of the algorithm's recommendation by reducing the insulin pump power, resulting in evident hyperglycemia. In Figure 4e, the glucose concentration increased and reached ≈10 mm at around the 16^th^ hour. When the algorithm started to calculate the recommended dose, we deliberately increased the GRI dose to 120% of the algorithm's recommendation, leading to observable hypoglycemia (rather than hyperglycemia) at around the 17^th^ hour. The timing of insulin delivery is also crucial. As shown in Figure 4f, insulin delivery within the recommended time slots achieved better control of glucose levels compared to administration at other time points outside the suggested time window. These findings highlight the critical role of precise insulin dosing and optimal timing in adaptive and reliable diabetes management. Insulin administration following the algorithm's recommendations, maintained glucose levels within the normoglycemic range, whereas deviations in either dose or timing led to significant dysregulation accompanied by hyperglycemia or hypoglycemia.

### 2.5 DuoLoop System Integration and Wearable Validation for Safety Enhancement

**Figure 5:**
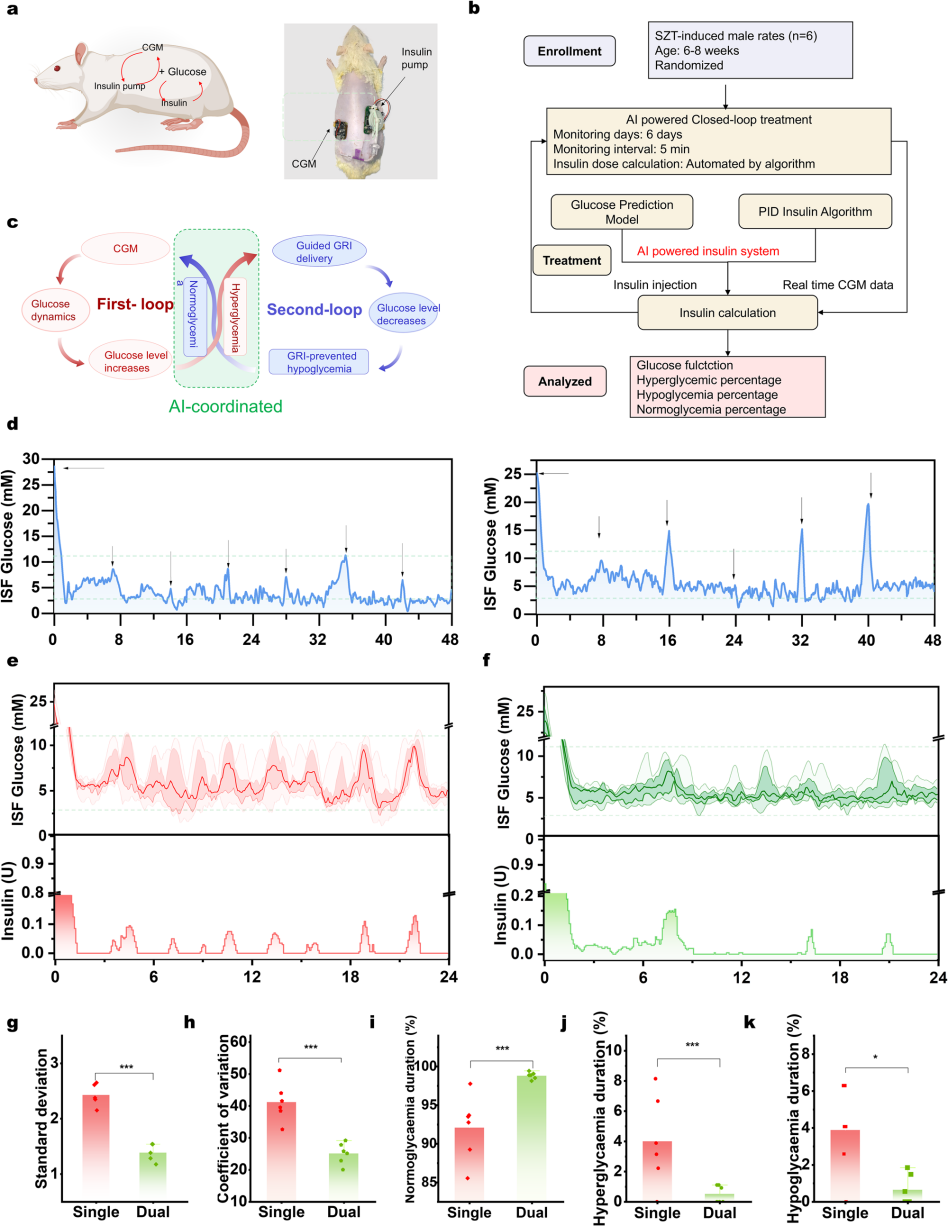
DuoLlLoop system integration and wearable validation for safety enhancement. a) Schematic (left) and actual images (right) of the validation of DuoLoop in a type 1 diabetic rat model. b) Trial profile of animal experiments. c) The edge‐AI‐powered dual closed‐loop system. d) Pure chemical closed‐loop treatment of diabetic rats through regular GRI injections (5 U/kg) administered every (left) 7 hours and (right) 8 hours. Comparisons of the combinational ISF glucose levels and the corresponding algorithm‐controlled insulin injection profiles in diabetic rats treated with e) SinLoop and f) DuoLoop. The glucose curves from top to bottom represent the 95%, 75%, 50%, 25%, and 5% intervals, respectively. Analysis of g) standard deviation, h) coefficient of variation, i) normoglycemia percentage, j) hyperglycemia percentage, and k) hypoglycemia percentage in diabetic rats treated with SinLoop (red) and DuoLoop (green). Initial insulin injection was administered to bring blood glucose to a normal level (typically within 1.5 hours). Statistical analysis includes data collected only after 1.5 hours.

The effectiveness of our wearable DuoLoop system in reducing the safety risk was validated in a type 1 diabetic rat model. This integrated system, which combines a CGM and an insulin pump, was placed on the rats' back and wirelessly controlled via Bluetooth, ensuring minimal interference with their daily activities (**Figure** **5**a,b). The operation of the DuoLoop system is outlined in Figure 5c. When the CGM detects an upward glucose trend nearing a hyperglycemic state, the algorithm activates the insulin pump to administer an insulin dose within a designated time frame. During hyperglycemia, insulin delivery is accelerated, and once glucose levels return to normoglycemia, the GRI release rate decreases to sustain stable glucose levels and prevent hypoglycemia.

The DuoLoop insulin delivery system demonstrated superior regulation efficiency compared to its single closed‐loop counterparts. First, we compared the DuoLoop system with a single chemical closed‐loop system (separated GRI injection). In brief, GRI was administered in 5 U doses (≈15 U/kg) every 7–8 hours to mimic a chemical‐only closed‐loop system (Figure 5d). Although glucose levels generally remained within the normoglycemic range, remarkable hyperglycemia and hypoglycemia still occurred. This finding suggests that although GRI can regulate insulin release in response to glucose fluctuations, the lack of precise, AI‐guided injections could lead to serious outcomes in diabetic subjects.

We then compared glycemic profiles between the single electrical closed‐loop system and our DuoLoop system (Figure S16, Supporting Information; Figure 5e,f). The single electrical closed‐loop system was built using a similar procedure as the DuoLoop system, but with RHI injection for data training. Visual observation toward the glucose curves indicates that single electrical closed‐loop system required multiple daily injections, while the DuoLoop system required fewer due to the longer normoglycemic duration achieved by GRI at equivalent doses (as previously shown in Figure 2e). Additionally, the DuoLoop system exhibited reduced glucose fluctuations compared to the traditional system, reflected in a smaller SD (2.44 vs 1.42) and CV (25.14 vs 41.22, Figure 5g,h). Clinical research has revealed that minimizing glucose fluctuations is essential for effective diabetes management.^[^ ^43^ ^]^ Thanks to the self‐regulating insulin delivery feature of GRI, the DuoLoop system maintained a higher percentage of normoglycemic periods (98.82% vs 92.10%, calculated from 1.5 to 24 h, the same below) with fewer instances of hypoglycemia (0.65% vs 3.89%) and hyperglycemia (0.52% vs 4.01%) than the traditional system (Figure 5i–k). It is important to note that both the SinLoop (with RHI) and DuoLoop (with GRI) systems were implemented using the same AI prediction model and PID controller. Therefore, the observed enhancements in glucose regulation with the DuoLoop system cannot be attributed to differences in the underlying control algorithm. Instead, these improvements arise from the dual‐loop configuration with the GRI. This distinction highlights the critical role of the dual‐loop design and the GRI in achieving superior system performance. Additionally, hematoxylin and eosin (H&E) staining and Masson's trichrome staining of injection sites revealed no significant neutrophil infiltration or collagen fiber formation (Figure S17, Supporting Information), confirming the biocompatibility and safety of the DuoLoop system for in vivo applications.

## 3 Conclusion

We have presented a wearable DuoLoop insulin delivery system to address the safety issues associated with traditional single closed‐loop ones. The 1^st^ closed‐loop system automates insulin delivery through wearable CGMs. The 2^nd^ closed‐loop system involves the GRI, where the insulin release rate depends on real‐time in vivo glucose levels. To link the two sections, an AI algorithm was developed by training on extensive glucose data sets, which enables accurate predictions and guides GRI delivery at optimal dosage and timing. In vivo validation demonstrated that the DuoLoop system significantly reduced glycemic fluctuations and occurrence of hyperglycemia and hypoglycemic compared to the traditional SinLoop system.

Despite these advantages, some limitations persist. One challenge lies in dietary management. Diet is a crucial factor in diabetes management.^[^ ^44^ ^]^ However, the diet of diabetic rats in our current rat model was unrestricted. Another limitation concerns the clinical applicability of DuoLoop. The current models are developed based on data from diabetic rat models, and future research should validate the system on other diabetic animals and, eventually, humans. Additionally, the long‐term stability and reliability of DuoLoop should be carefully evaluated over extended periods. Overall, the DuoLoop presented in this work marks an advancement towards precision diabetes management. We envision that the proposed dual or multiple closed‐loop device concept could be adopted by other wearable systems to regulate metabolic processes in other diseases, addressing broader clinical needs.

## 4 Experimental Section

### Materials

PEDOT:PSS aqueous suspension (Clevios PH1000) was purchased from Heraeus Electronic Material (USA). Glycerol, dodecylbenzene sulfonic acid (DBSA), sodium chloride, (3‐glycidyloxypropyl) trimethoxysilane (GOPS), calcium chloride (CaCl_2_), and 3‐(trimethoxysilyl) propyl methacrylate (TMSPMA) were purchased from the Sigma‐Aldrich (USA). D‐glucose, GOx, glutaraldehyde, and ferrocene, chitosan, and acetic acid were provided by Aladdin Co. (Shanghai, China). The polyimide (PI) thin film was obtained from the DuPont Co. (U.S.A.). The biocompatible adhesive polyurethane was provided by 3M (U.S.A.). The chemicals were used without further purification unless otherwise specified.

### Fabrication of GRI

The GRI was prepared by integrating a polymer, PEG‐PLL‐FPBA, with RHI through electrostatic interactions. To fabricate the polymer, PEG5000 (51.2 mg) and PLL (MW = 30–70 kDa, 54.9 mg) were dissolved in water and stirred overnight at a pH of 7.3. Subsequently, a solution of FPBA‐NHS (58.4 mg) in DMSO was added to the mixture and allowed to react for an additional 30 minutes. The final product was obtained through lyophilization, yielding 109.6 mg (66.62%). Next, polymer and RHI were consistently combined at a mass ratio of 10:1 to fabricate GRI. First, 10 mg of polymer material was dissolved in 1 mL of DI water. Separately, 10 mg of RHI was dissolved in 1 mL of an acidic solution (60 µL of 1 m HCl in 1 mL DI water). Equal volumes of the polymer and RHI solutions were then mixed, and the pH of the mixture was promptly adjusted to 7.4 using sodium hydroxide.

### OECT Fabrication

The PEDOT:PSS ink for the channel material was prepared as follows: pristine PEDOT:PSS ink was first mixed with GOPS (1 w/w.%), glycerol (5 v/v.%), and DBSA (0.1 v/v.%) and stirred with a vortex mixer for 3 minutes. The resulting suspension was then filtered through a polytetrafluoroethylene (PTFE) membrane (0.45 µm pore size) to remove aggregates and prevent nozzle clogging. The gate, source, and drain electrodes were fabricated by depositing a thin layer of gold on a PI thin film using mask‐assisted e‐beam deposition. The PEDOT:PSS channel layer was deposited between the source and drain electrodes by inkjet printing and then dried at 110 °C for 15 minutes. A UV‐cured resin was applied as an insulating layer. The hollow microneedles to extract ISF were fabricated using a high‐resolution 3D printing system (microArch S240). After printing, the microneedles were UV‐cured to improve strength and durability.

### Fabrication of Glucose Gate Electrode

First, 30 mg of glucose oxidase (GOx) was dissolved in 1 mL of PBS solution. Then, 50 mg of chitosan and 50 µL of acetic acid were dispersed in 10 mL of DI water, stirred at 500 rpm for 12 hours at 60 °C. Afterward, 1 mL of the GOx‐PBS solution was added to 1 mL of the chitosan solution and sonicated for 20 minutes prior to use.^[^ ^45^ ^]^ For the ferrocene solution, 18.6 mg of ferrocene was dissolved in 10 mL of ethanol. Then, 4 µL of the ferrocene solution was deposited onto the gate electrode and dried at room temperature for 1 hour. Next, 4 µL of the GOx/chitosan mixture was applied to the gate electrode and dried at 4 °C for 3 hours.^[^ ^46^ ^]^ Finally, the glucose‐sensing gate electrode was fabricated by applying glucose‐sensing solution on the pristine gate electrode.

### The Basic Framework of the Algorithm

The first part of the Edge‐AI involves a blood glucose prediction algorithm, comprised of a Transformer network. This network processes real‐time CGM data along with historical records. The input L_1_ is fed into the encoder, while input L_2_, along with zero‐padding B_3_, is fed into the decoder structure. The network's output, spanning a future time range t, provides predicted blood glucose variations within the length of B_3_. The blood glucose prediction Transformer network is quantized for deployment on mobile devices. The predicted results are then transmitted to the insulin pump for insulin dosage adjustments. The second part comprises the GRI injection algorithm, a sustainable adaptive PID control algorithm. This algorithm regulates appropriate insulin injection dosages by utilizing current CGM data and the target blood glucose value. Additionally, it incorporates predicted future trend variations as input to assist in adjusting the rate parameters of the PID controller, which can be directly deployed on the insulin pump for edge computations.

### Model Building

The core of the Transformer Encoder‐Decoder architecture lies in the self‐attention module and the positional linear projection network. DuoLoop leverages the Encoder and Decoder to process long‐range and short‐range CGM signals, respectively. This mechanism enables DouLoop to focus on both long‐term and short‐term modeling of CGM signals, thereby enhancing its ability to predict future glucose levels and facilitating the precise release of insulin during delivery.

The Encoder module utilizes multi‐head self‐attention to process long‐range CGM information. Given a historical CGM signal of length *m*, the long‐range signal *L*_1_ of length *n* is extracted and fed into the multi‐head self‐attention module:

(3) $$Q_{en}=W_{q}\cdot L_{1},K_{\mathrm{en}}=W_{k}\cdot L_{1},\;\;V_{\mathrm{en}}=W_{v}\cdot L_{1}$$

where $W_{q}\in R^{d_{q}\times h\times d}$, $W_{k}\in R^{d_{k}\times h\times d}$ and $W_{v}\in R^{d_{v}\times h\times d}$ denote the learnable Query, Key and Value matrix in attention core, *h* represents the number of attention heads. Finally, the attention computation is given as:

(4) $${O_{en}}^{i}=Softmax\left(\frac{{Q_{\mathrm{en}}}^{i}\cdot {K_{\mathrm{en}}}^{i\;T}}{\sqrt{d}}\right){{V^{\prime }}_{\mathrm{en}}}^{i}$$

(5) $$H_{en}=O_{en}+V_{en}$$

where $O_{en}^{i}$ represents the output of the *i^th^* attention head, and  *V*′_en_ denotes the Value matrix after normalization. Subsequently, the attention output is combined with the initial Value matrix *V_en_* through a residual connection to obtain the long‐range CGM modeling output of the Encoder, denoted as *H_e_*.

In the Decoder, the attention computation mechanism is essentially the same as Equation (3). The input to the Decoder is *L*_2_, a masked multi‐head self‐attention mechanism is employed, which yields:

(6) $${O_{de}}^{i}=Softmax\left(\frac{Mask\left({Q_{de}}^{i}\cdot {K_{de}}^{i\;T}\right)}{\sqrt{d}}\right){{V^{\prime }}_{de}}^{i}$$

(7) $$H_{de}=O_{de}+V_{de}$$

where 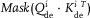 represents the application of a triangular mask to the scores computed between the Key and Value matrices. Specifically, the upper triangular portion of the score matrix is masked with negative infinity. This ensures that future information is not accessed during decoding. In the second module of the Decoder, a cross‐attention mechanism is employed to combine the long‐range CGM signals modeled by the Encoder with the short‐range CGM signals from the Decoder. This integration is achieved using the following equation:

(8)

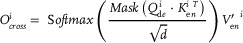

(9) $$H_{cross}=\mathrm{FFN}\left(O_{de}\right)+V_{de}$$

In the cross‐attention computation, the Query is derived from the Decoder, while the Key and Value are obtained from the Encoder. Finally, the model's prediction results are produced through a Feed‐Forward Network (FFN) layer.

### Model Training

The study implemented the model using PyTorch 2.0.1 and trained it on NVIDIA GTX 2080 Ti GPUs. The training process utilized the Adam optimizer and was conducted 30 epochs. To prevent overfitting and enhance generalization, an early stopping mechanism with a patience value of 5 was applied. Mean Squared Error (MSE) was chosen as the standard loss function for model optimization.

### Model Compression

To efficiently deploy the neural network‐based blood glucose prediction algorithm, quantization compression was utilized to reduce the storage size of the model. To verify the predictive accuracy of the quantized model, neural network quantization techniques were employed as follows:

(10) $$W_{int}=round\;\left(\frac{W_{float}}{s}\right)$$

(11) $$W_{q}=clamp\;\left(-2^{b-1},2^{b-1}-1.\;W_{int}\right)$$

where *W_float_* denotes the original 32‐bit weight matrix in glucose Transformer, *s* represents the scale factor, which maps the 32‐bit matrix to integers. And *b* denotes the quantization bit‐width (e.g., 4/8), *W_q_* is the weight matrix post‐quantization. Specifically, the original 32‐bit model was compressed to 8‐bit or even 4‐bit, achieving a compression rate of over three times. This significantly reduces the computational cost of deploying neural networks while maintaining accurate predictive performance and ensuring system security (Table S2, Supporting Information).

### Validation of the Algorithm on a UVA/Padova Simulator

The fluctuations in blood glucose levels from CGM data and real‐time computed insulin injection doses were simulated over a 24‐hour period under random dietary intake. With the support of the closed‐loop algorithm, the blood glucose levels of simulated diabetic patients were effectively maintained within the target range. To evaluate the algorithm's performance, extensive blood glucose variation data were collected over 30 days from eight virtual patients. The Transformer‐based blood glucose prediction model was trained using 24 days (80%) of data from these virtual patients, while the remaining six days of data were used for testing. The figure presents a continuous sequence of 1000 predicted test data points for a single patient. The red curve represents the algorithm's predicted values, whereas the black curve illustrates the actual blood glucose variations generated by the simulator.

### General Animal Experiment Protocol

The animal procedures were approved by the Animal Ethics Committee of Guangzhou Medical University (protocol no. GY2023‐590). Male Sprague‐Dawley (SD) rats (300–350 g, aged 6–8 weeks) were obtained from the Guangdong Medical Laboratory Animal Center. 6–8‐week‐old male SD rats were selected because this age provides stable metabolic parameters and suitable sensitivity to STZ, ensuring reliable induction of hyperglycemia. Before experiments, all animals were acclimated for one week under standardized, pathogen‐free conditions at the Laboratory Animal Center of Guangzhou Medical University. Type 1 diabetes was induced in the rats by a single injection of STZ (110 mg kg^−1^) following a 12‐hour fast. Diabetic rats had free access to food and water throughout the experiment unless otherwise specified. They were housed in a controlled environment with an ambient temperature of 20–26 °C and relative humidity of 50–70%. Diabetic rats were divided into groups and given subcutaneous injections of RHI or GRI. Efforts were made to minimize animal suffering, including the use of isoflurane anesthesia, and to reduce the number of animals used in the study.

### Intraperitoneal Glucose Tolerance Test

Diabetic rats were administered either RHI or GRI at a dose of 25 U/kg. Two hours later, all rats received an intraperitoneal injection of glucose (0.3 g mL^−1^ in PBS, 1.5 g kg^−1^). ISF glucose levels were continuously monitored and analyzed thereafter.

### Histopathological Study

The diabetic rats received subcutaneous injections of either PBS (control group) or GRI (experimental group, 25 U/kg, n = 3) on days 1, 3, 5, and 7. Subcutaneous tissue sections from the injection sites were extracted, fixed in 4% paraformaldehyde, embedded in paraffin, and then sliced into thin sections. H&E and Masson's trichrome staining images were obtained using a digital slide scanner (VS200, Olympus) and analyzed with Olympus Image Viewer software.

### Statistical Analysis

All results are presented as means ± standard deviation (SD). Two tailed unpaired Student's *t*‐test was used for comparisons between two groups. Differences between experimental and control groups were considered statistically significant at *p* < 0.05. In this study, significance levels are indicated as *p < 0.05, **p < 0.01, ***p < 0.001, and ****p < 0.0001.

## Conflict of Interest

A US Patent has been filed for this work (US Application No. 63/746,228).

## Author Contributions

S.Z. and J.W. conceived this project. S.Z. acquired funding and supervised the whole research. X.H., W.H., W.L., and S.L. conducted experiments and collected the data. W.H. contributed to the algorithm design and simulation. J.W. fabricated the GRI. X.H., W.L., and J.Z. designed and performed animal experiments. B.C., X.T., J.B., D.L., I.P., and H.H. helped process the data. S.Z., X.H., and W.H. drafted the manuscript. All authors contributed to the revision of the manuscript.

## Supporting information



Supporting Information

## Data Availability

The data that support the findings of this study are available from the corresponding author upon reasonable request.
