# Supplementary material for: A Wearable, Dual Closed‐loop Insulin Delivery System for Precision Diabetes Management
Source: Adv Mater. 2026 Jan 12;38(8):e14945. doi: 10.1002/adma.202514945 (PMC12879279; doi:10.1002/adma.202514945)
Supplement: Supplementary file 1 — Supporting Information [file ADMA-38-e14945-s001.pdf]

ADVANCED MATERIALS

Supporting Information

for *Adv. Mater.*, DOI 10.1002/adma.202514945

A Wearable, Dual Closed-loop Insulin Delivery System for Precision Diabetes Management

Xuecheng He, Wei Huang, Wensheng Lin, Binbin Cui, Xinyu Tian, Jing Bai, Dingyao Liu, Ivo Pang, Hao Huang, Shixian Lin, Jixiang Zhu, Jinqiang Wang* and Shiming Zhang**

ADVANCED MATERIALS

Supporting Information

for *Adv. Mater.*, DOI 10.1002/adma.202514945

A Wearable, Dual Closed-Loop Insulin Delivery System for Precision Diabetes Management

Xuecheng He, Wei Huang, Wensheng Lin, Binbin Cui, Xinyu Tian, Jing Bai, Dingyao Liu, Ivo Pang, Hao Huang, Shixian Lin, Jixiang Zhu, Jinqiang Wang* and Shiming Zhang**

Supporting Notes

Supporting Note 1. The necessity of development of an OECT-Microneedle as an auxiliary CGM sensor. We use a combination of commercial (Sinocare) CGMs and a customized OECT device for continuous glucose monitoring in ISF. Commercial CGMs offer essential glucose monitoring; however, their effective detection range is limited to 2-25 mM. When glucose levels exceed this range, the commercial device merely indicates “25 mM” or “2 mM” without specifying the actual concentration. While this range suffices for regular monitoring, precise glucose levels are needed for a more accurate and reliable algorithm. To address this issue, we have developed a microneedle-based OECT as an auxiliary tool to enhance the CGM’s functionality and improve the accuracy of glucose detection[1].

Supporting Note 2. Transformer Prediction Model and PID system for DuoLoop System. In our experiments, the proposed DuoLoop model demonstrated the ability to accurately predict blood glucose dynamics for virtual patients and rats over the next 30 minutes. This predicted trend better accurately reflects the current rate of glycemia dynamics. Such predictions implicitly model the residual insulin levels and glucose metabolism rates in rats[2]. Therefore, we believe that accurate predictions can contribute to optimizing real-time insulin injection dosages.

Specifically, we reformulated the traditional PID system's method of modeling the glucose change rate, which relies solely on historical data, $dG/dt = (Gt - G(t-1))/5$, into an average rate of change that incorporates both historical and predicted data[3]. By integrating the Transformer model, we enhanced this with a more precise predictive capability using $dG/dt = (G\_(t+6) - G\_(t-6))/(6*5)$. Predicting future trends enables a more accurate reflection of the current rate of glucose change.

Supporting Note 3. Clarke Error Grid for evaluation of glucose prediction model. The Clarke Error Grid (CEG) is a widely used tool for evaluating the clinical accuracy of glucose prediction models[4]. It divides the prediction-actual glucose plot into five regions:

Region A: Predictions are clinically accurate, either within ±20% of the actual glucose or both values are in the hypoglycemic range. Predictions in this region are ideal and do not affect treatment decisions.

Region B: Predictions deviate slightly but remain clinically acceptable, posing no significant risk to patient safety.

Region C: Predictions show significant deviation, leading to potential misinterpretation of glucose levels and inappropriate clinical decisions.

Region D: Predictions are dangerously inaccurate and could result in incorrect treatment, such as mistaking hypoglycemia for hyperglycemia or vice versa.

Region E: Predictions are entirely unrelated to actual glucose values and are clinically unacceptable.

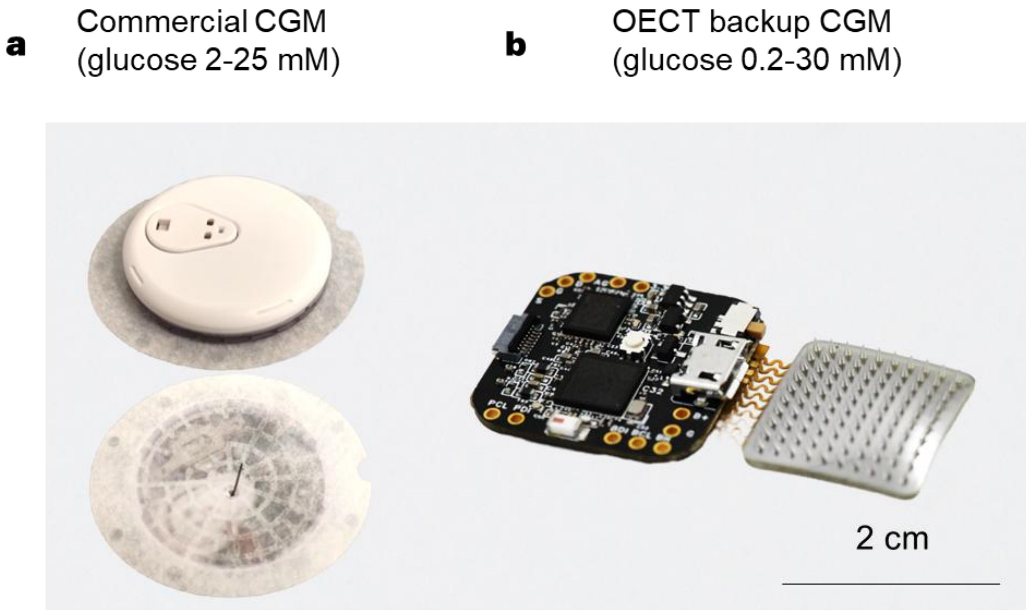

Figure S1. Illustrations of commercial and OECT backup CGMs. (a) Optical images of commercial CGMs, which cannot glucose levels beyond 2-25 mM. (b) Optical images of the customized OECT-CGM connected to a readout system, which can extend the detective glucose range, serving as a complementary backup to commercial CGMs.

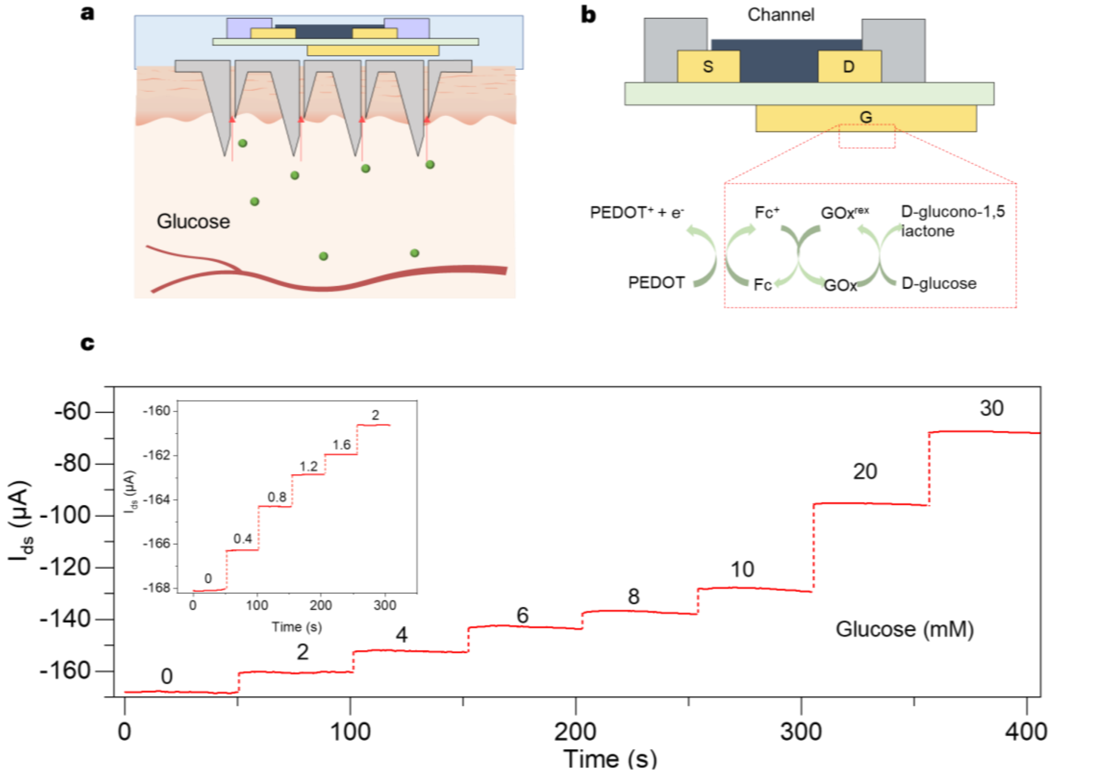

Figure S2. Glucose detection using the OECT backup CGM. (a) Schematic of glucose diffusion mechanics between the ISF and the OECT via the hollow microneedle. (b) Schematic of the OECT-based glucose sensing mechanism, where the GOx-modified gate electrode catalyzes glucose decomposition. This reaction, facilitated by a ferrocene mediator, promotes electron transport at the gate electrode, resulting in a sensitive and glucose-dependent variation in current (ΔI_{ds}) between the source and drain electrodes. (c) Real-time current response (ΔI_{ds}) of the OECT-based sensor to glucose concentrations ranging from 0 to 30 mM. The inset highlights the detection of low glucose concentrations (0-2 mM).

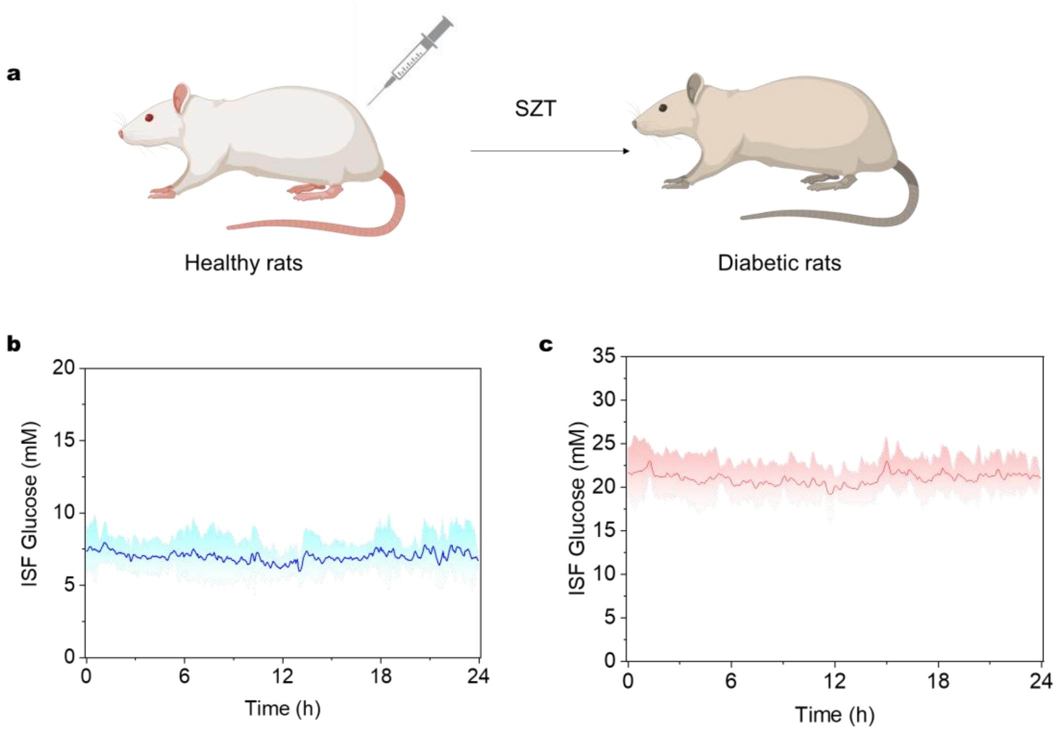

Figure S3. Construction of type 1 diabetic rats. (a) Schematic representation of STZ injection in rats. Glucose dynamics in (b) healthy rats and (c) diabetic rats without insulin treatment.

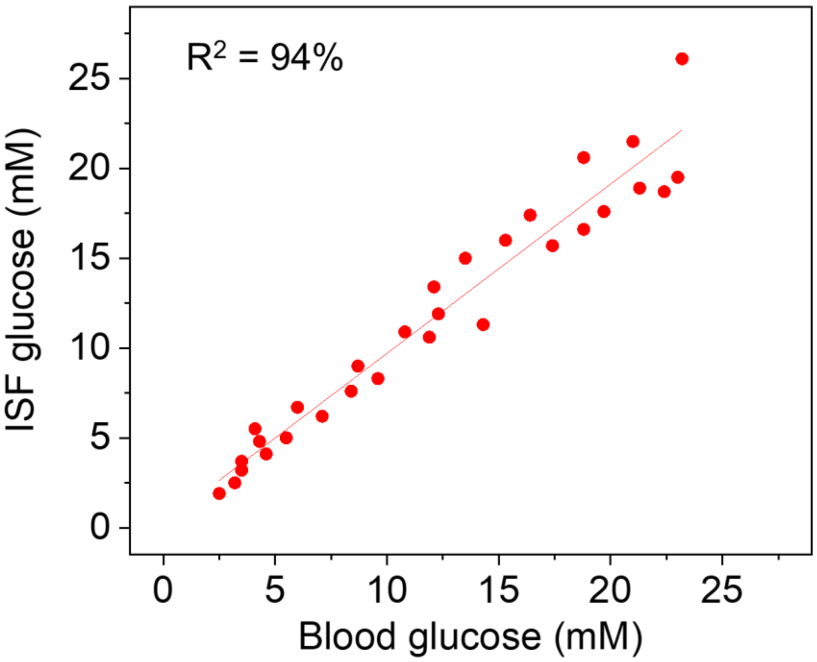

Figure S4. One-to-one correlation between blood glucose and interstitial fluid glucose levels (n=31).

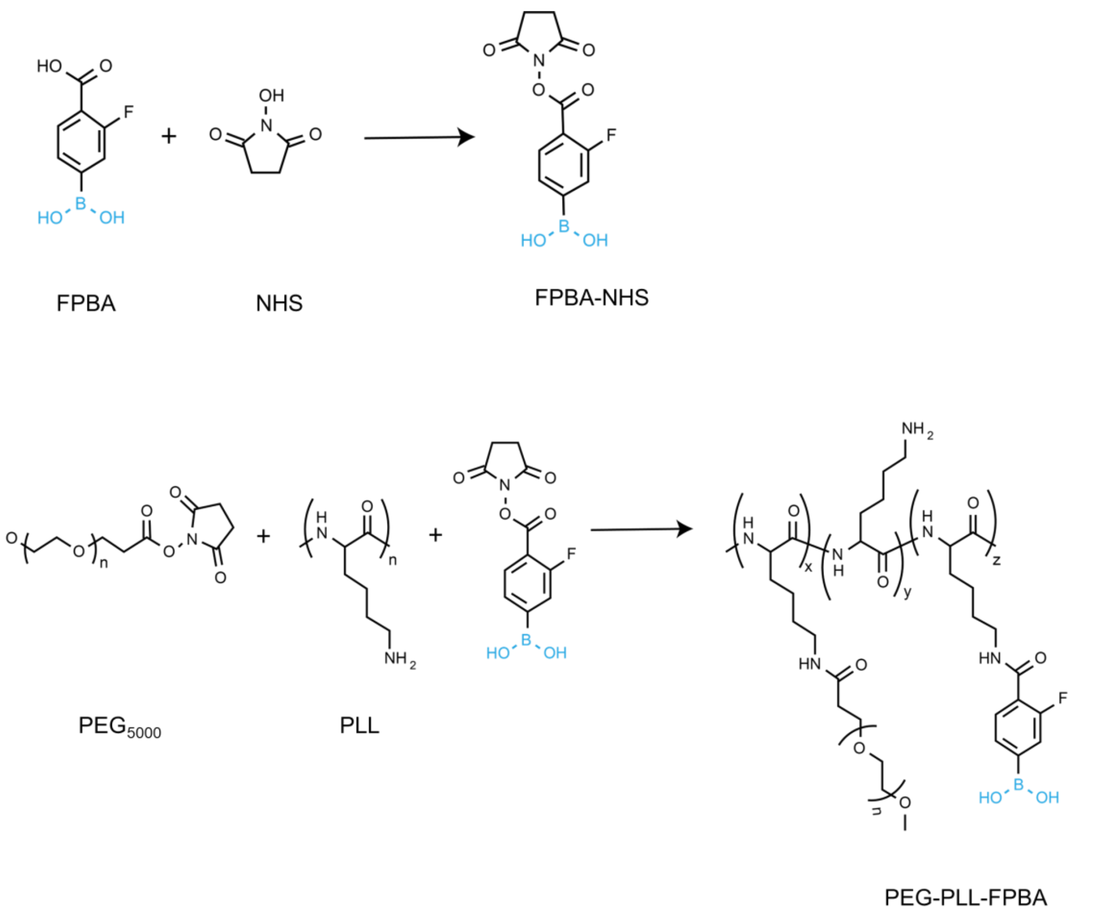

Figure S5. The synthesis route of PEG-PLL-FPBA (PPF).

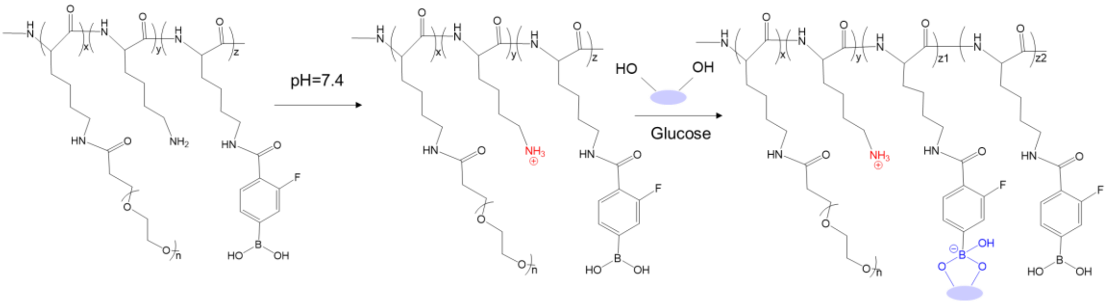

Figure S6. The chemical structure of the PEG-PLL-FPBA and the reaction mechanism with glucose. The hydrolytic equilibrium of phenylboronic acid in the presence of glucose facilitates functional interaction under hyperglycemic conditions. The FPBA moieties bind to the diol groups of glucose molecules, introducing negative charges that subsequently trigger insulin release.

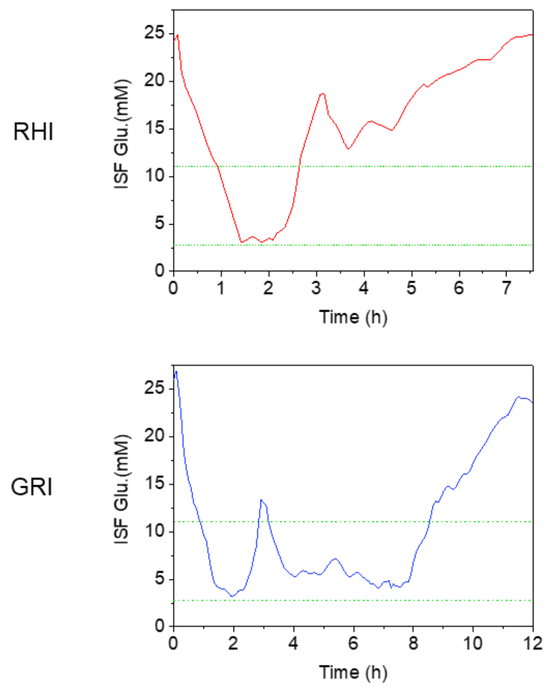

Figure S7. In vivo glucose tolerance test in diabetic rats 2 hours after RHI or GRI injections.

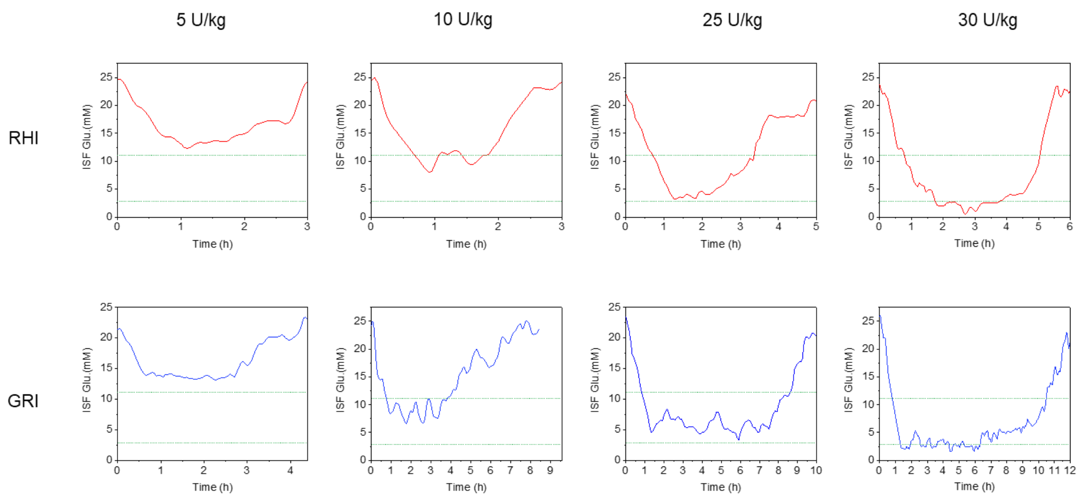

Figure S8. Glycemic profiles of diabetic rats treated with varying doses of RHI and GRI.

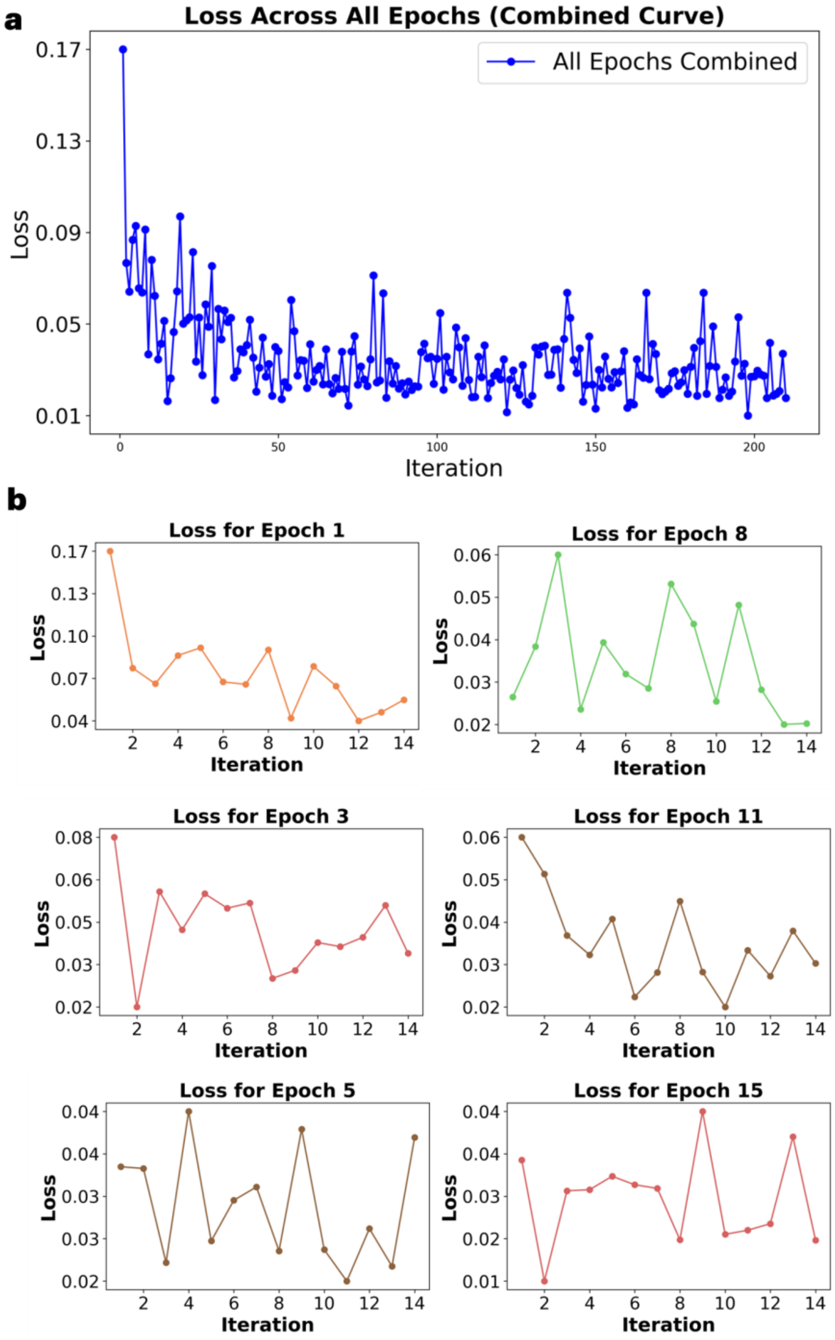

Figure S9. The training loss curves, depicting the performance and convergence behavior of glucose prediction for virtual patients. (a) Training loss on the UVA/Padova simulator for eight virtual patients, with a rapid decline during the first 50 iterations, followed by stabilization, indicating effective learning and model convergence within 15 epochs. (b) Detailed view highlighting variability in loss reduction across virtual patients during selected epochs, reflecting individual differences in glucose dynamics.

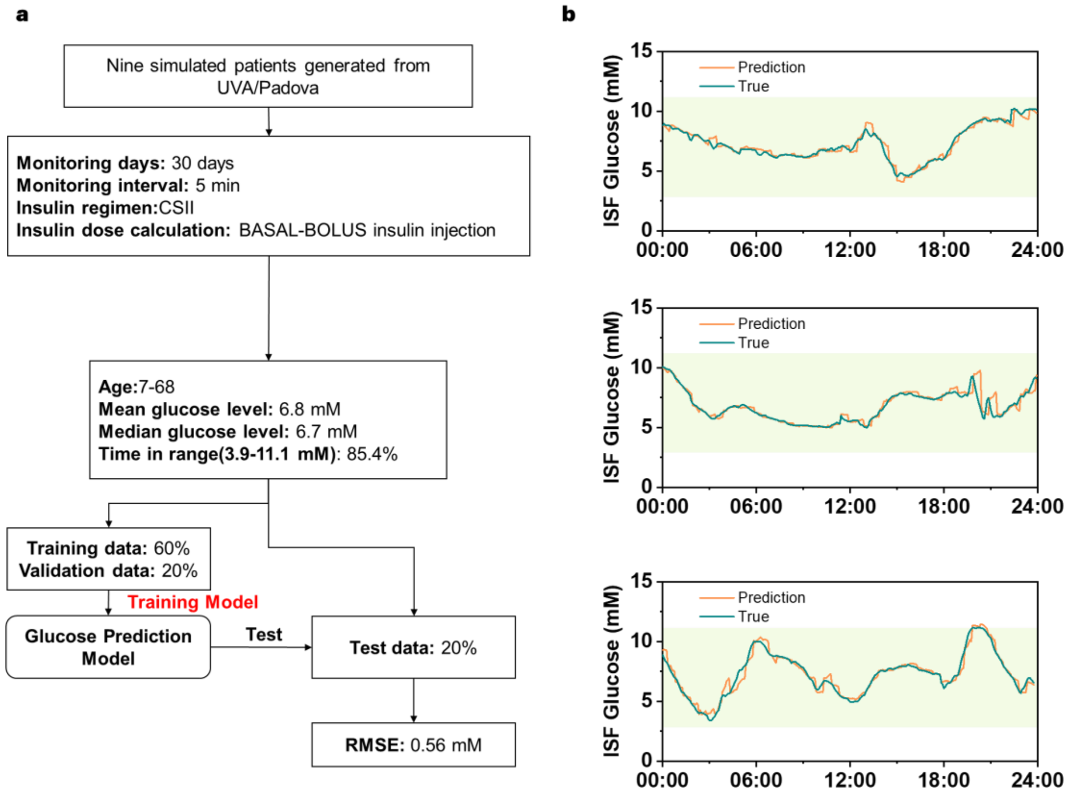

Figure S10. Validation of the AI-powered insulin delivery system by integrating glucose prediction model and PID insulin algorithm on simulated virtual patients. (a) Flow diagrams of the glucose prediction Transformer model. (b) Predicted and true glucose profiles of the simulated virtual patient. RMSE: root mean square error.

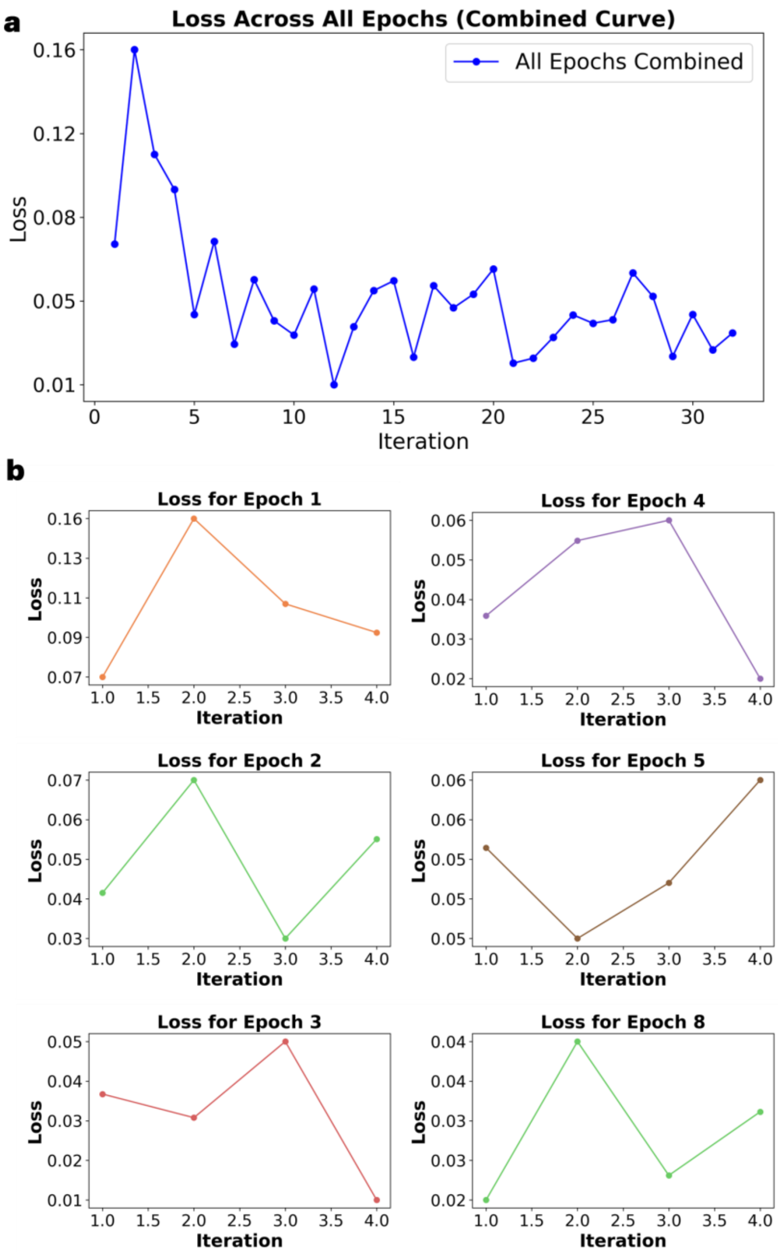

Figure S11. Training loss curves illustrating the performance and convergence behavior of glucose prediction for experimental rats. (a) Training loss for 10 experimental rats, with a steep decline within the first 10 iterations and stabilization by the end of 8 epochs, demonstrating rapid convergence and effective adaptation to smaller, heterogeneous datasets, highlighting the feasibility of transfer learning techniques. (b) Loss trajectories for selected epochs, showing consistent learning across individual rats.

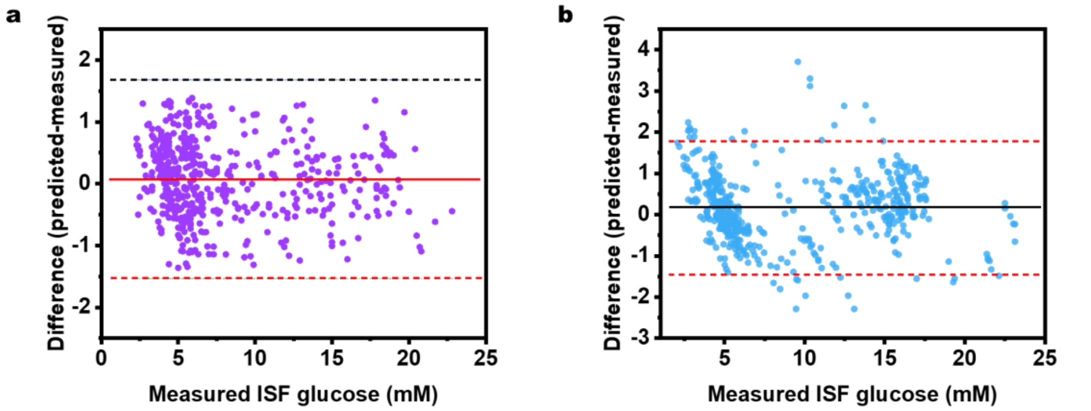

Figure S12. The Bland–Altman plot showing agreement between the predicted and measured ISF glucose. (a) The in-domain test set, where a mean difference of 0.17 mM is observed, and all the data points are within 95% limits of agreement defined by ±1.96 S.D. (b) The out-domain test set, where a mean difference of 0.07 mM is observed, and 486 of 516 (94.19%) data points are within 95% limits of agreement defined by ±1.96 S.D.

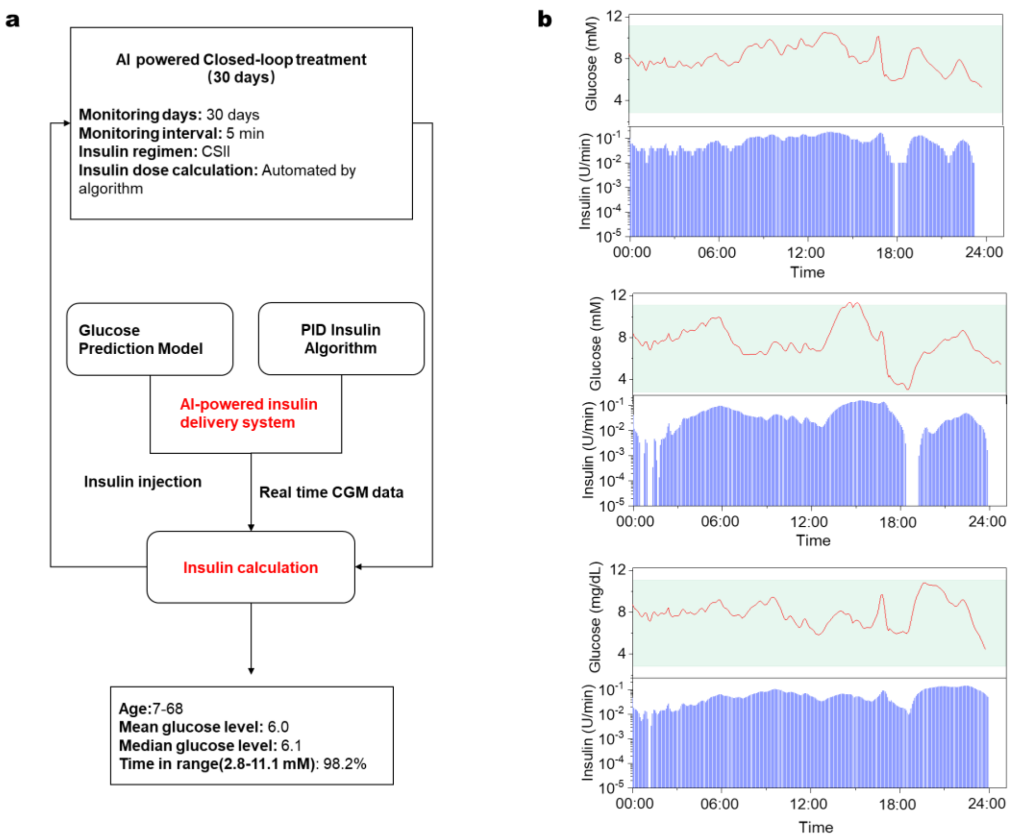

Figure S13. Validation of the AI-powered insulin delivery system by integrating glucose prediction model and PID insulin algorithm on simulated virtual patients. (a) General flow diagrams. (b) Glucose curves of simulated patients using the integrated glucose prediction model and PID insulin algorithm, with most glucose levels falling within the normoglycemic range, indicating its effectiveness.

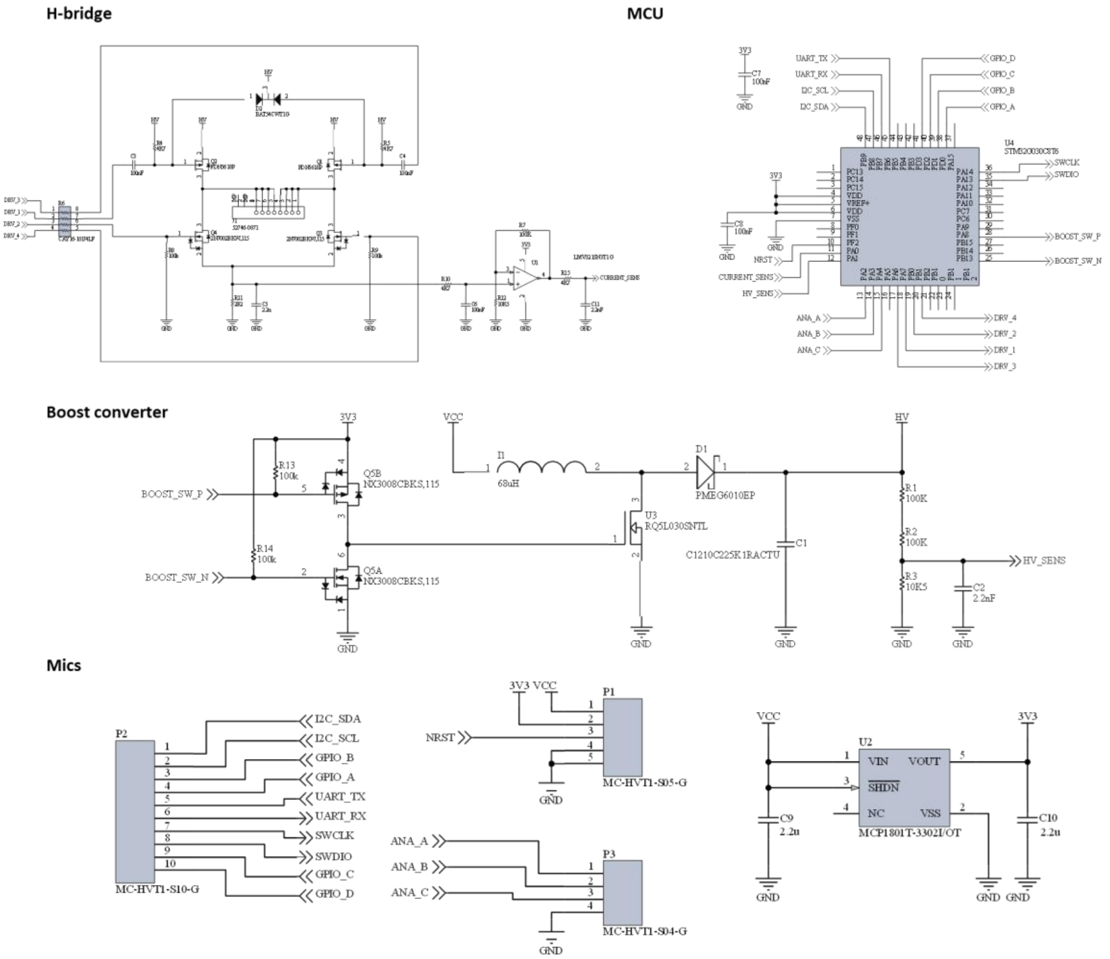

Figure S14. Detailed circuit schematic of the insulin pump.

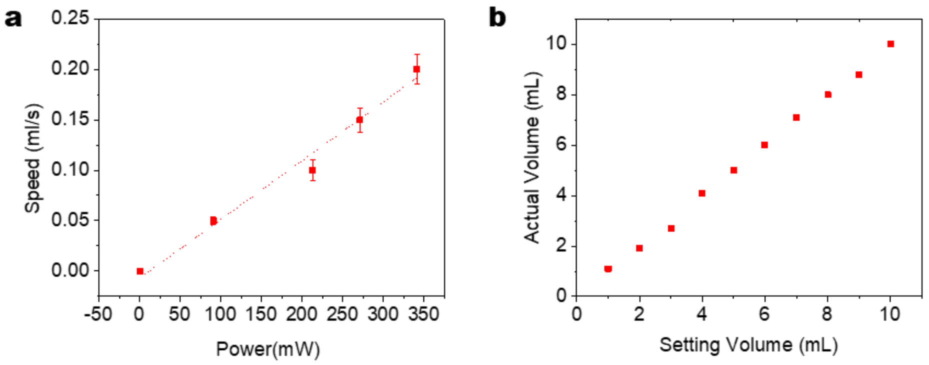

Figure S15. Characterizations of the customized insulin pump. (a) Correlation between pump power and solution delivery speed. (b) Consistency between the actual delivered volume and the preset volume of the customized insulin pump.

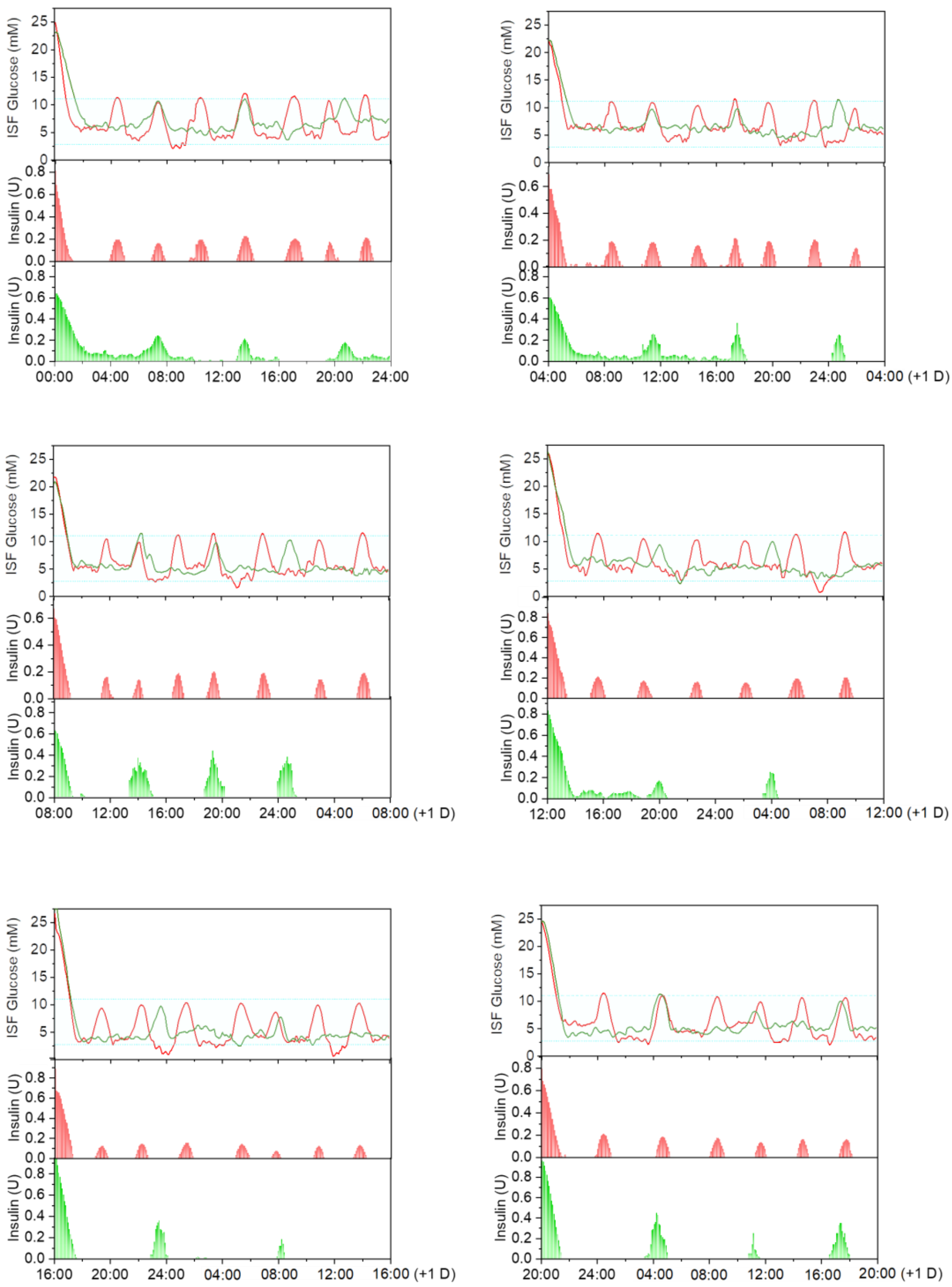

Figure S16. Comparisons of ISF glucose levels in diabetic rats treated with SinLoop (red) and DuoLoop (green) systems. Histograms represent the corresponding insulin injection.

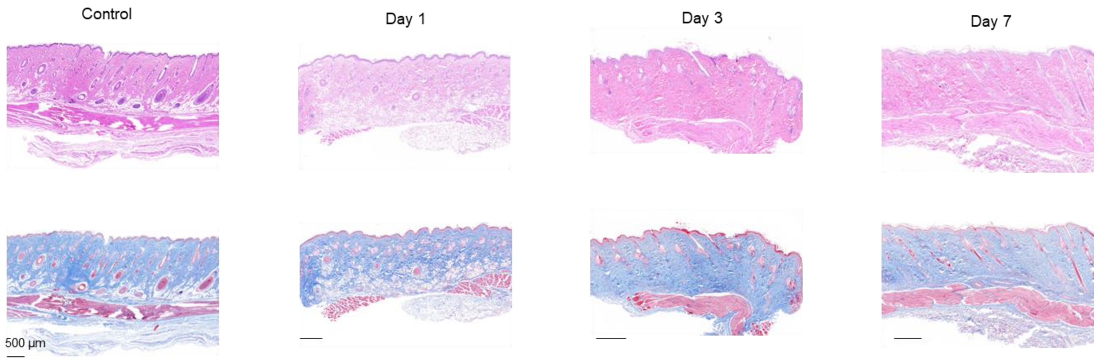

Figure S17. In vivo biosafety characterization. Representative images of Hematoxylin & Eosin (H&E, top) and Masson's trichrome (MTC, bottom)-stained sections of the full-thickness insulin delivery site over 7 days of treatment.

Supporting Table 1 The Transformer model structure size and parameter count for blood glucose prediction. The developed blood glucose prediction model employs an Encoder-Decoder architecture, where the Encoder consists of a single multi-head self-attention layer with a head size of 8 and a hidden dimension of 128. The Decoder comprises two multi-head self-attention layers with a head size of 8 and a hidden dimension of 128. The final model outputs the predicted blood glucose results through a prediction layer.

Model Arch.	General Framework	Operation Name	Layer Name	Hidden Dimensions
Transformer	Encoder	Multi-Head Self-Attention layer (head = 8)	Q	256
			K	
			V	
			Out	
	Decoder	Multi-Head Self-Attention layer (head = 8)	Q	256
			K	
			V	
			Out	
		Multi-Head Self-Attention layer (head = 8)	Q	
			K	
			V	
			Out	
		Prediction layer	Projection	

Supporting Table 2 The comparison for the PID control results with/without the Transformer.

Patient Number	RMSE (mM)	TIR (w Transformer)	TIR (w/o Transformer)
Patient 1	0.57	96.7%+9.5%	87.2%
Patient 2	0.43	98.3%+6.8%	85.5%
Patient 3	0.62	98.4%+11.9%	86.5%
Patient 4	0.33	97.2%+10.1%	87.1%
Patient 5	0.54	98.6%+11.8%	86.8%
Patient 6	0.47	95.9%+9.0%	86.9%
Patient 7	0.30	98.5%+11.2%	87.3%
Patient 8	0.33	96.3%+9.9%	86.4%
Patient 9	0.49	98.2%+11.2%	86.6%
Patient 10	0.52	97.8%+9.2%	87.0%

Supporting Table 3 Compressed model performance with quantization for mobile inference. The 32-bit model represents the full-precision training model, indicating the initial training results. The 8-bit and 4-bit models represent quantized compressed models with reduced weight bit-widths, tailored for deployment on edge mobile devices.

Bit-width	Model Size (Mb)	RMSE-30 minute (mM)	Compression Ratio
32 bit	7.6	10.01	-
8-bit	2.1	9.87	72.34%
4-bit	1.2	11.24	84.21%

Supporting References:

[1] J. Bai, D. Liu, X. Tian, Y. Wang, B. Cui, Y. Yang, S. Dai, W. Lin, J. Zhu, J. Wang, A. Xu, Z. Gu, *and* S. Zhang, *Sci. Adv.* **2024**, *10*, eadl1856.

[2] A. B. Daly, C. K. Boughton, M. Nwokolo, S. Hartnell, M. E. Wilinska, A. Cezar, M. L. Evans, *and* R. Hovorka, *Nat. Med.* **2023**, *29*, 203.

[3] A.-L. Alshalalfah, G. B. Hamad, *and* O. A. Mohamed, *IEEE Trans. Circuits Syst. I: Regul. Pap.* **2021**, *68*, 3147.

[4] W. L. Clarke, D. Cox, L. A. Gonder-Frederick, W. Carter, *and* S. L. Pohl, *Diabetes Care* **1987**, *10*, 622.
